# Dynamic functional connectivity in borderline personality disorder: associations with trauma, emotion regulation and symptom severity

**DOI:** 10.1007/s00406-026-02255-5

**Published:** 2026-05-08

**Authors:** Arshia Rana, Marie-Luise Otte, Giovanni Fazio, Daniele Olivio, Yéléna Le Prieult, Mike M. Schmitgen, Yunus Balcik, Mert Koc, Chantal Tech, Nadine D. Wolf, Fabio Sambataro, Robert Christian Wolf

**Affiliations:** 1 Department of General Psychiatry, Center for Psychosocial Medicine, Heidelberg University, Voßstraße 2, 69115 Heidelberg, Germany; 2 Department of Neuroscience (DNS), University of Padova, Padova, Italy; 3 German Center for Mental Health (DZPG) partner site Mannheim/Heidelberg/Ulm (ZIHub), Mannheim, Germany

**Keywords:** Borderline personality disorder, Chronnectome, Resting state, Magnetic resonance imaging, Cluster-states, Meta-states, Time-varying connectivity

## Abstract

**Background:**

Altered intrinsic functional connectivity is a well-established marker of borderline personality disorder (BPD). However, recent research suggests that investigating brain dynamics may offer a more detailed perspective on the neural signatures of BPD-related symptoms.

**Methods:**

Resting-state fMRI data were analyzed in female patients with BPD (*n* = 47) and healthy controls (*n* = 28) to derive dynamic functional network connectivity (dFNC) indices using both meta-state and cluster-state approaches. Between-group comparisons assessed BPD-related dFNC alterations, while dimensional analyses explored associations between network dynamics and distinct symptom dimensions.

**Results:**

Both meta-state and cluster-state analyses revealed strong associations between symptom dimensions and dynamic range and fluidity. Meta-state analysis indicated that greater emotion regulation difficulties corresponded to an expanded state repertoire, reflecting increased variability in large-scale network configurations. Cluster-state analysis showed that fewer state transitions were associated with heightened borderline symptom severity, greater childhood trauma exposure, and increased dissociative symptoms. Furthermore, childhood trauma and emotion regulation difficulties moderated the relationship between time spent in specific cluster-states and borderline symptom severity.

**Conclusion:**

These findings suggest that reduced dynamic flexibility but increased dynamic range in large-scale brain networks may contribute to core BPD symptoms, particularly in early trauma, emotion dysregulation as well as borderline symptom severity. The results highlight the association between aberrant dFNC and BPD and contribute to a more detailed characterization of neural dynamics relevant to the disorder.

**Supplementary Information:**

The online version contains supplementary material available at 10.1007/s00406-026-02255-5.

## Introduction

Borderline personality disorder (BPD) is characterized by pervasive instability in interpersonal relationships, self-image, and emotion regulation (ER), alongside pronounced impulsivity [1]. It is associated with significant psychosocial impairment, placing a considerable burden on healthcare resources across various treatment settings [2, 3]. The disorder’s complexity is further compounded by high rates of comorbidities with other mental health conditions, including depression, anxiety, dissociative disorders, and impulse-related disorders such as substance use and eating disorders [4].

Environmental risk factors for BPD are well-documented, with adverse childhood experiences—such as physical, sexual, and emotional abuse, attachment disruptions, childhood psychopathology, and dysfunctional parenting—playing a pivotal role in its etiology [5, 6]. The neurobiological underpinnings of BPD, however, remain a subject of ongoing investigation. It has been proposed that childhood trauma, in conjunction with inherited temperament traits, may modulate gene expression through neuroplastic alterations in brain structure and function, contributing to the emergence of persistent affect regulation impairments and personality traits characteristic of BPD [7]. While genetic studies linking specific polymorphisms to BPD and early adversity have yielded inconsistent meta-analytical support [8], neuroimaging research has provided more compelling evidence for altered neural functioning in BPD [9]. Resting-state functional connectivity (rs-FC) is widely employed in neuroimaging research to examine spontaneous fluctuations in brain activity. By measuring interregional statistical dependencies in the absence of task demands, rs-FC provides insights into the intrinsic organization of large-scale neural networks and has been associated with behavioral and clinical phenotypes, including personality disorders [10, 11]. However, traditional rs-FC studies have largely operated under the assumption that connectivity remains static throughout the scan duration. Subsequent research has demonstrated that functional interactions between brain regions fluctuate over time in a neurobiologically meaningful manner [12], necessitating a shift towards dynamic functional network connectivity (dFNC) analyses.

Analyses of functional connectivity through a dynamic lens allow for the examination of temporal variations in connectivity, thereby offering a more nuanced understanding of neural network organization [13, 14]. Among the various methods used to study resting-state dynamics, the sliding-window approach is the most widely applied [15]. Two prominent analytical frameworks in dFNC research are the cluster-state and meta-state approaches. The cluster-state method characterizes connectivity states as discrete, recurrent patterns and tracks their transitions over time, assuming that an individual occupies only one state at any given moment [12, 16]. In contrast, the meta-state approach aggregates connectivity profiles across network pairs, providing a broader perspective on the extent of engagement across multiple states simultaneously [17].

Recent fMRI studies have demonstrated the potential of dFNC metrics as biomarkers of human cognition and behavior [18, 19]. This methodology has been extensively applied to psychiatric disorders, including schizophrenia [20], depression [21], Attention-Deficit/Hyperactivity Disorder (ADHD) [22], cannabis use disorder [23], and autism [24], though investigations into personality disorders such as BPD remain limited. Notably, a recent study on cocaine use disorder found that diminished inter-network connectivity correlated with higher impulsivity and borderline trait severity, suggesting that dynamic dysconnectivity may underlie BPD-related psychopathology [25]. Additionally, research has linked alterations in brain dynamics to neuroticism [26], a core feature of BPD [27], as well as to childhood maltreatment [28] and emotion dysregulation [29]—both of which are highly prevalent among individuals with BPD. Despite these promising findings, direct investigations of brain dynamics in BPD remain scarce.

Given the potential of dFNC as a marker of maladaptive traits and its capacity to yield novel insights [19], the present study sought to expand our understanding of functional network dynamics in BPD by examining their associations with core symptom dimensions. Specifically, we hypothesized that individuals with BPD would exhibit disrupted connectivity patterns relative to healthy controls. Moreover, symptom dimensions such as ER difficulties, depression, dissociation, and impulsivity were expected to demonstrate aberrant relationships with dFNC. To achieve a thorough characterization of neural dynamics, both meta-state and cluster-state dFNC approaches were employed, providing a broad and integrative view of connectivity alterations in BPD.

## Methods

### Participants

The sample consisted of 79 participants including those with a current diagnosis of borderline personality disorder (BPD), recruited from various treatment settings and with the use of flyers, online communications as well as advertisements, and Healthy Controls (HC). Out of the 79 participants, 4 were excluded due to missing data before the data analyses were commenced. The final sample consisted of 75 participants (47 BPD and 28 HC). In order to avoid the confounding effects of neurological differences, only female individuals [30] between the ages of 18–60 were included in the study. Of the 47 participants with BPD, 19 experienced symptoms of auditory voice hearing not related to substance abuse and were recruited as part of a broader DFG-funded research project. The general exclusion criteria were identifying as male or diverse, left-handedness, the presence of current and lifetime neurological disease or current medical condition known to affect brain function as well as acute (self-)aggression, acute suicidal ideation and claustrophobia. Furthermore, persons with insufficient German language skills, pronounced formal thought disorder and general contraindications for MRI were excluded from participation in the present study. Moreover, healthy controls were required to report the life-long absence of mental illness as well as that of first-degree relatives with mental illness. For participants with BPD, additional exclusion criteria included comorbidities such as a history of or current schizophrenia spectrum disorders, psychotic disorders as part of other medical conditions, moderate substance abuse disorder (excluding nicotine) until 3 months prior to the enrolment, and a severe depressive episode with psychotic symptoms. A minimum period of two weeks of stable psychotropic medicinal regimes without major adjustments and a diagnosis of BPD according to DSM-V criteria was required for admission in the study.

The following demographic data was collected: age, gender, years of education, as well as current medications and current and lifetime comorbidities. Medications taken by individuals with BPD included antidepressants, antipsychotics, mood stabilizers, stimulants, sedatives and other common medicines such as thyroid medication, asthma sprays, and oral contraception. History of comorbid current and life-time mental disorders included depressive disorders (*n* = 43), PTSD (*n* = 17), attention disorders (*n* = 6), dissociative disorders (*n* = 6), social phobia (*n* = 3), generalized anxiety disorder (*n* = 2), lifetime obsessive-compulsive disorders (*n* = 3), lifetime eating disorders including anorexia and bulimia nervosa (*n* = 4) and amphetamine dependence (*n* = 1). To preserve clinical representativeness, participants with psychiatric comorbidities alongside BPD were not excluded from the study. Moreover, one of the participants in the BPD group reported a short period of drowsiness/sleeping during the resting state fMRI measurement and was not excluded from the analyses. Among the various psychometric assessments administered, the following clinical measures were analyzed: depressive symptoms using the Hamilton Rating Scale for Depression (HAM-D) [31], emotion regulation difficulties via the Difficulties in Emotion Regulation Scale (DERS) [32], impulsivity through the Barratt Impulsiveness Scale (BIS-11) [33], and dissociation using the German adaptation of the Dissociative Tension Scale, the *Dissoziations-Spannungs-Skala* (DSS) [34]. Additionally, childhood trauma severity was assessed with the Childhood Trauma Questionnaire-Short Form (CTQ-SF) [35], while borderline symptom severity was evaluated using the short version of the Borderline Symptom List-95, the Borderline Symptom List-23 (BSL-23) [36].

### MRI data acquisition

A Siemens MRI PRISMA system at 3 Tesla made available by the Department of Neuroradiology at Heidelberg University was used for the present study. Structural data was collected using a T1-weighted (3D) magnetization prepared rapid gradient echo (MP-RAGE) sequence with the following parameters: flip angle = 9°, echo time (TE) = 2.26 ms, repetition time (TR)=1900 ms, inversion time (TI) = 900 ms, FOV = 256 mm, slice plane = S > T-3.9 > C0.8, slice thickness=1 mm, voxel size = 1.0 × 1.0 × 1.0, distance factor = 50%,number of slices = 176. The rs-fMRI images were obtained using a multiband-T2*-weighted echo-planar-imaging (EPI) sequence according to the Minnesota cmrr-protocol with the following parameters: TE = 30.00 ms, TR = 2000 ms, flip angle = 70°, field of view (FOV) = 192 mm, slice plane = transversal, voxel size = 3.0 × 3.0 × 4.0 mm, distance factor = 25%, PAT factor = 3, number of measurements = 200.

### MRI data analysis

**Fig. 1:**
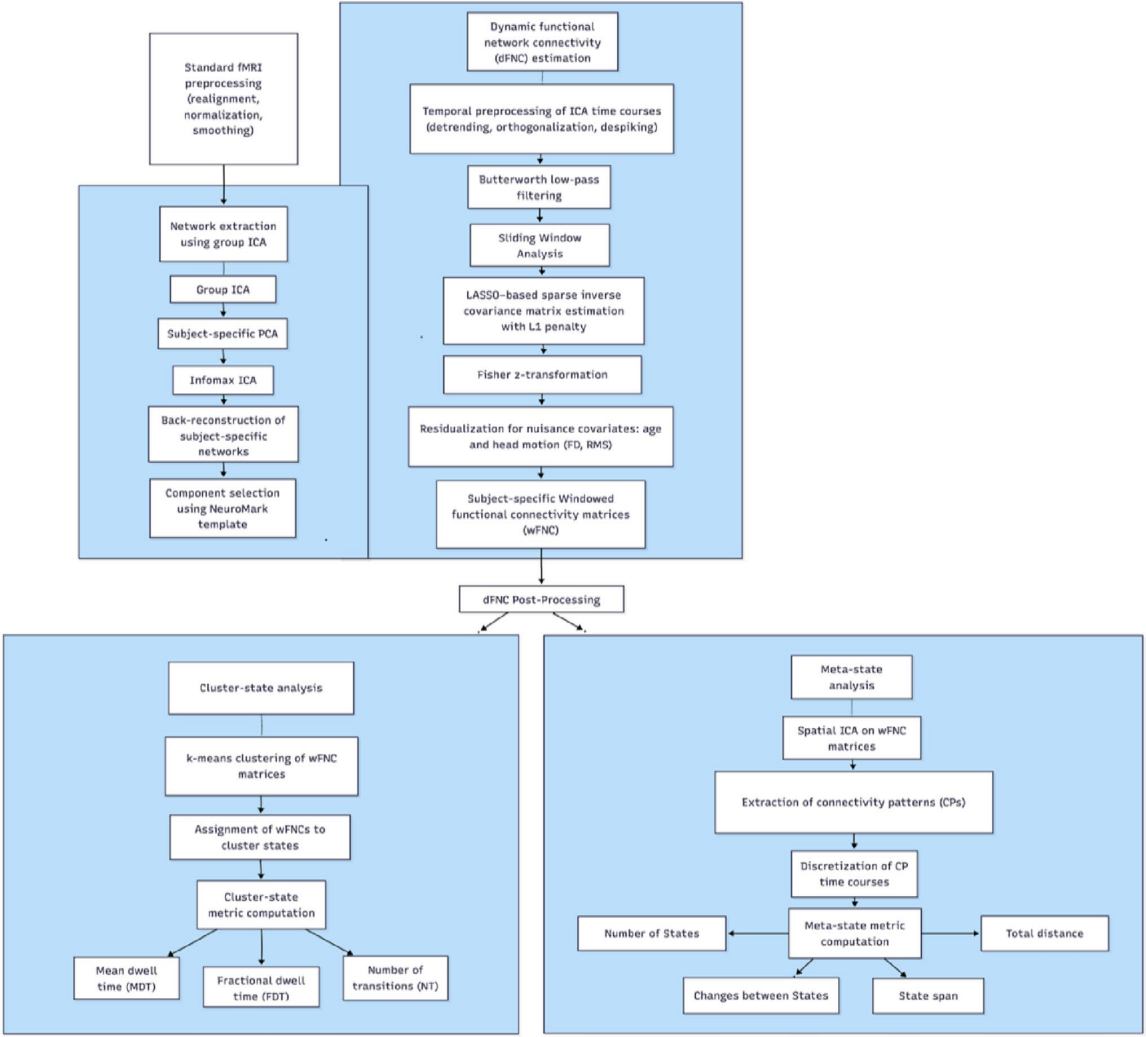
Schematic Representation of Dynamic Functional Connectivity Data Analysis Process. The schematic illustrates the dynamic functional network connectivity (dFNC) analysis pipeline applied to resting-state fMRI data. Cluster-state analysis resulted in state-based metrics including Mean Dwell Time (MDT), Fractional Dwell Time (FDT), and Number Of Transitions (NT). In parallel, meta-state analysis yielded higher-order connectivity dynamics, including Number Of States, Changes Between States, State Span, and Total Distance. *FD* Framewise displacement, *RMS* Root mean square, *PCA* Principal component analysis, *ICA* Independent component analysis, *wFNC* Windowed functional connectivity matrices, *CP* Connectivity pattern

After the removal of the initial 8 scans to allow for subject-state stabilisation, preprocessing of the data was carried out using the Data Processing Assistant for rs-fMRI (DPABI/DPARSF 5.2) [37]. This was followed by a slice timing correction against temporal inconsistency, reorientation, and realignment for head movement correction. After co-registering the functional and structural images, they were segmented and normalized on a Montreal Neurological Institute (MNI) template using DARTEL [38]. Nuisance covariates were not regressed at this stage. For the final step of smoothing, a 6-mm 3D Gaussian kernel full width at half maximum (FWHM) with a default mask was used. All further data analysis steps pertaining to the dFNC pipeline can be seen in a schematic visualization in Fig. 1 below.

The group ICA of fMRI toolbox (GIFT) (https://trendscenter.org/software/gift/*)* [39] was used to reduce the data into a number of functional networks. To identify these intrinsic connectivity networks, a spatial group independent component analysis was performed, including subject-specific Principal Component Analysis (PCA) to decompose subject-specific data, and further reduction into independent components (ICs) using the infomax algorithm. This was followed by a back-reconstruction of subject-specific spatial maps and the respective time courses using a group information guided-ICA (GIG-ICA) algorithm for constrained ICA on the NeuroMark template. This template comprises of 53 intrinsic connectivity networks that were grouped into 7 resting-state networks: subcortical (SC), auditory (AU), sensorimotor (SM), visual (VI), cognitive control (CC), default mode (DM) and cerebellar (CB) [40]. Using the ICASSO toolbox, the ICA process was repeated 50 times in order to ensure reliable estimation [41].

### Dynamic functional connectivity

Estimates of dFNC were calculated using the sliding window analysis in the GIFT toolbox [16]. The 53 time courses of each intrinsic connectivity network (ICN) were detrended, orthogonalized and despiked using 3Despike (https://afni.nimh.nih.gov/pub/dist/doc/program_help/3dDespike.html). Next, a fifth-order Butterworth low-pass filter was applied to filter these time courses [42]. Using a Least Absolute Shrinkage and Selection Operator (LASSO) regression [43] a sparse inverse covariance matrix, of 53 × 53 was obtained in line with the number of ICNs to construct functional connectivity networks, with an additional L1 penalty to improve sparsity. The L1 Regularization was repeated 10 times. A Gaussian alpha value of 3 and window size of 30 TR was used with a sigma of 3 TR and a 1 TR increment-step, resulting in 160 windows. A Fishers-Z transformation was carried out on the resulting matrices and residualized to nuisance covariates comprising age, head motion operationalized as mean framewise displacement (FD) [44], as well as the root mean square (RMS) of head motion. As a result, windowed correlation matrices were obtained for each subject, along with the dFNC parameters. The dFNC post-processing was two-fold, including the meta-state and cluster-state analyses.

### Meta-state analysis

In the meta-state analyses of dFNC, windowed FNCs (wFNC) are modelled as maximally independent connectivity patterns (CP), where a discretized vector of CP weights is extracted from each wFNC. This vector is known as a meta-state [17]. This approach helps view the functional dynamics of network-pair correlations at an aggregated level, which can deepen the understanding of disorders that manifest themselves across complex symptom categories and behavior patterns.

In this study, a spatial ICA (sICA) was used to decompose each wFNC into maximally independent prototype components or connectivity patterns (CPs) using the INFOMAX algorithm. The number of CPs were considered to be 5, in line with existing literature, to achieve a balance of including complex linearly additive effects as well as retaining a richly featured basis pattern (10 repetitions in ICASSO) [16, 17]. The time courses of these CPs were obtained by regressing each participant’s dFNC data at each time window on the group of sICA CPs. In order to work with a more manageable space, the values of time courses of the CPs were discretized into 8 bins of positive and negative quartiles that each corresponded to a meta-state [17]. Thus, a five-dimensional time-coursed vector was obtained, where time courses changed at each level. According to meta-state calculations, each participant could possibly be in more than one meta-state at one point in time. This time-dependent distribution of weighted probability helps represent statistical measures of meta-state dynamism. Following this procedure, a total of 4 metrics were computed for each participant to explore these dynamics. These included the total number of meta-states occupied during the scan (Number Of States), and total number of switches between meta-states (Change Between States), as indicators of dynamic fluidity, and the maximum L1 distance between two distinct meta-states occupied by the participants (State Span) and total distance travelled by each participant across the state space (Total Distance), as indicators of dynamic range [17].

### Cluster-state analysis

In the following framework, a cluster-based approach was used to analyse state-based dFNC. For this purpose, k-means clustering of wFNC was repeatedly implemented to reduce all dynamic connectivity to a sequence of a few states. The number of states was 3 as determined by the elbow criterion [45]. Each wFNC was assigned to one of the states that the wFNC resembled the most. This method allowed us to reduce a space of over 1000 dimensions into one, thereby resulting in the most recurring connectivity pattern. A participant will thus be in one state at any given time point [16]. For each state the Mean Dwell Time (MDT) spent in a specific state by each participant was computed, measured in the number of consecutive windows [16]. In addition, the Fraction Of Dwell Time (FDT), i.e. the total scanning time spent dwelling in a specific state and the total Number Of Transitions (NT) from one state to another were obtained for each participant.

### Statistical analyses

All statistical analyses were performed using JASP, a R-based statistical interface [46]. Skewness and kurtosis values with thresholds of ± 1.96 [47] and ± 7 [48] respectively as well as visual inspection of histograms were used for testing normality assumptions. Two-tailed t-tests and Mann- Whitney-U tests were used to compare HCs to participants with BPD regarding the meta-state and cluster-state dFNC indices as well as head motion parameters. These indices were later correlated with clinical scores of HAM-D, BIS-11, BSL-23, CTQ, DSS and DERS questionnaires in the BPD group, using Spearman rank correlation analyses. Moreover, exploratory linear regressions were computed to evaluate the moderating role of ER difficulties and childhood trauma severity in the association between borderline symptom severity and FDT of connectivity states. Two linear regression models were used for each moderation analysis, with and without the interaction term respectively, to evaluate the increase in explained variance. Additionally, pairwise Spearman rank correlations were computed to examine associations between dFNC indices, clinical symptom measures, and quality control variables within the BPD group as well as for the combined sample including participants of both BPD and HC groups. A nominal significance level of *p* < 0.05 was chosen for all analyses.

### Post-hoc sensitivity analyses

As part of a sensitivity analysis framework, all primary statistical analyses were repeated under two conditions: (1) introducing covariate control of key variables, and (2) excluding influential points while controlling for the same covariates as in the first condition. Key covariates included in both scenarios were: age, head motion i.e. FD and medication (sum of z-scored olanzapine and fluoxetine dose equivalents). Influential points that were excluded in the second condition were – (a) identified motion outliers (FD > 0.3 mm) and (b) one BPD participant who reported temporary drowsiness during the fMRI scan. First, within-group correlation analyses between dFNC metrics and clinical scores in the BPD group were re-estimated under both conditions (1) and (2). Secondly, between-group differences were also reassessed under both conditions using two-tailed analyses of covariance (ANCOVAs).

Two moderation Analyses were repeated using robust linear regression (MM-estimation with Tukey’s bisquare influence function), while controlling for age, FD and medication. Lastly, a post-hoc sensitivity power analysis was conducted to contextualize the findings of the present study.

## Results

### Participants

The study included 47 participants with BPD and 28 HCs between the ages of 18 and 59. All participants were female and right-handed. All group differences in clinical scores of the BPD and HC groups were statistically significant, while differences in age and years of education were not (Table 1).

**Table 1:** Demographic and clinical characteristics of participants

	Group; Mean (SD)		Test statistic	*p*
	HC (*n* = 28)	BPD (*n* = 47)		
Age (years)	24.85 (5.44)	27.85 (8.01)	804.50	0.10 ^m^
Education (years)	16.00 (2.77)	15.63 (3.27)	506.00	0.67 ^m^
BSL-23	3.96 (5.05)	46.21(21.28)	1299.00	< 0.001^m^
HAM-D	0.89 (2.13)	12.63 (8.39)	1252.00	< 0.001^m^
BIS-11	55.67 (7.34)	73.42 (9.87)	8.23	< 0.001^t^
DSS	26.42 (47.93)	401.70 (327.40)	1250.00	< 0.001^m^
CTQ-SF	44.53 (9.34)	70.27 (19.46)	1192.00	< 0.001^m^
DERS	66.35 (16.95)	123.44 (22.18)	12.55	< 0.001^t^

Values are indicated as Mean (SD).

BSL-23 borderline symptom list-23, HAM-D Hamilton depression scale, BIS-11 barratt impulsiveness scale-11, DSS dissoziations-spannungs-skala, CTQ-SF childhood trauma questionnaire-short form, DERS difficulty in emotion regulation scale

^m^Results from Mann-Whitney U tests. ^t^Results from Student’s T-Test

Spearman rank correlations between cluster-state and meta-state dFNC indices, clinical symptom measures, and key covariates (age, medication, FD, and RMS) were examined within the BPD group (Supplementary Material: Fig. A) and across the combined sample including both HC and participants with BPD (Supplementary Material: Fig. B). Correlation coefficients were modest in magnitude and did not reach statistical significance, indicating minimal confounding effects.

Between-group analyses for head motion, assessed using Mann-Whitney *U* Tests did not yield statistically significant differences between BPD and HC groups. There were no group differences in RMS, *U* = 709.00, *p* = 0.582, rank-biserial *r* = − 0.08, 95% CI [− 0.34, 0.19], or FD, *U* = 600.00, *p* = 0.531, rank-biserial *r* = 0.09, 95% CI [− 0.18, 0.35] (Supplementary Material: Fig. C). However, three participants were found to have exceeded the motion threshold of FD > 0.3 mm. Of those, two belonged to the BPD group and one to the HC group. The three participants were removed in the subsequent exclusion-based condition of the post-hoc sensitivity analyses. (Supplementary Material: Table D2 and Table E2).

### Meta-state analysis

**Fig. 2:**
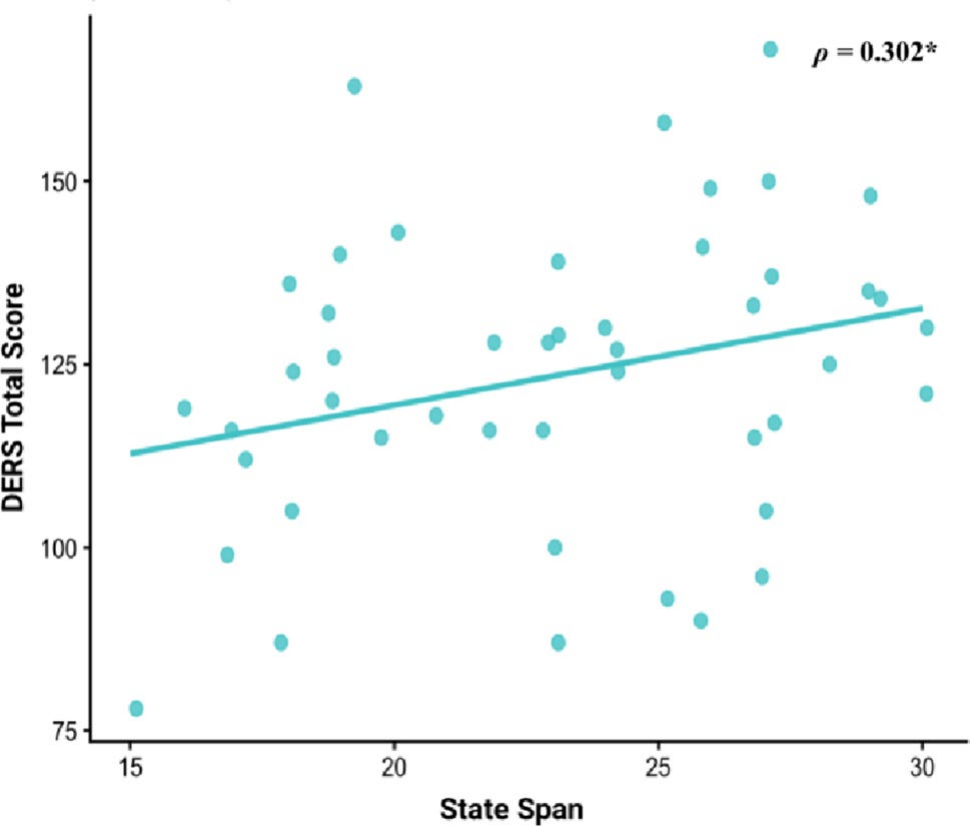
Association Between Emotion Regulation Difficulties and State Span in the BPD Group. *ρ* indicates Spearman’s rho; * indicates statistical significance with a *p *< 0.05. *DERS* Difficulty in Emotion Regulation Scale

No significant differences were observed in the meta-state dFNC indices between BPD patients and healthy controls (Supplementary Material: Table B). Within the BPD group, scores on the DERS rating scale were significantly positively correlated with the State Span (i.e., the maximum L1 distance between two distinct meta-states), *r*(45) = 0.302, *p* = 0.039 (Fig. 2). All other correlations between clinical scores and meta-state indices of dFNC were non-significant (Supplementary Material: Table A).

### Cluster-state analysis

**Fig. 3:**
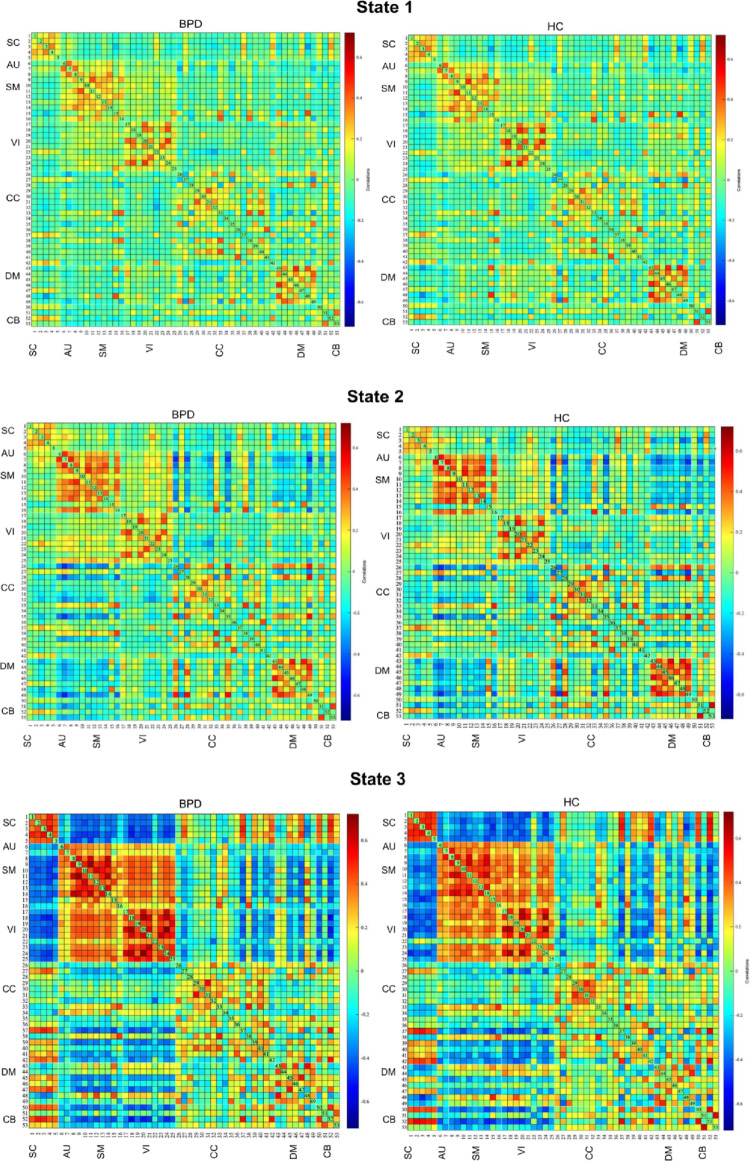
Connectivity Patterns of the Three Dynamic Functional Connectivity States identified using Cluster-state Analysis. The three most recurrent connectivity patterns identified using k-means clustering, with each state represented by distinct 53 × 53 matrices of the pairwise correlations between independent components. State-specific connectivity patterns are shown for both BPD (left) and HC groups (right), respectively. The x- and y-axes list the individual components. *SC S*ubcortical domain; *AU* Auditory domain; *SM* Sensorimotor domain; *VI* Visual domain; *CC* Cognitive control domain; *DM* Default mode domain; *CB* Cerebellum domain. Colour bar on the right indicates positive (warmer tones) and negative (cooler tones) correlations.

A total of 3 states were identified, with each state showing different patterns of dynamic connectivity (Fig. 3). For ease of interpretation, brief descriptive labels were assigned to each state (Supplementary Material: Table G).

#### State 1

State 1 occurred in 60.7% of time windows across all participants, marked by sparse correlations. It occurred for 45 participants with BPD and 26 HC. In this state, within-network correlations were highest in DM, VI and SM, and SC, though overall diminished in comparison to other states. Between-network correlations were highest in AU (negative with DM and CB) and SC (negative with SM, VI, and CC), SM (negative with DM and CB), CC (negative with SC, VI and some ICs of DM and CB). Between-network correlations between VI and SM were next to zero or very mildly positive.

#### State 2

Across all participants, this state occurred in 26.5% of the time windows. It occurred for 35 BPD participants and 20 HC, and showed denser functional network correlations, as compared to State 1. In this state, within-network correlations were highest in the AU, SM, DM and SC whereas the highest between-network correlations were found in SC (negative with CC, DM, CB), AU (negative with CC, DM, CB and positive with SM and some ICs of VI), SM (negative with CC, DM, CB and positive with VI), DM (negative with AU, SM and some ICs of SC, and positive as well as negative with some ICs of CC).

#### State 3

State 3 occurred in 12.8% of the time windows, for 9 BPD participants and 9 HC. As compared to the other two, this state was marked by very dense correlations between and within networks. The highest within-network correlations in this state were found in SC, SM, VI, and DM. Within-network correlations were high for SC (negative with AU, SM, VI and positive with CB, DM and some ICs of CC), AU (positive with SM and VI and negative with SC, DM, CB, and CC), SM (positive with VI, and negative with DM, CB, SC and some ICs of CC).

**Fig. 4:**
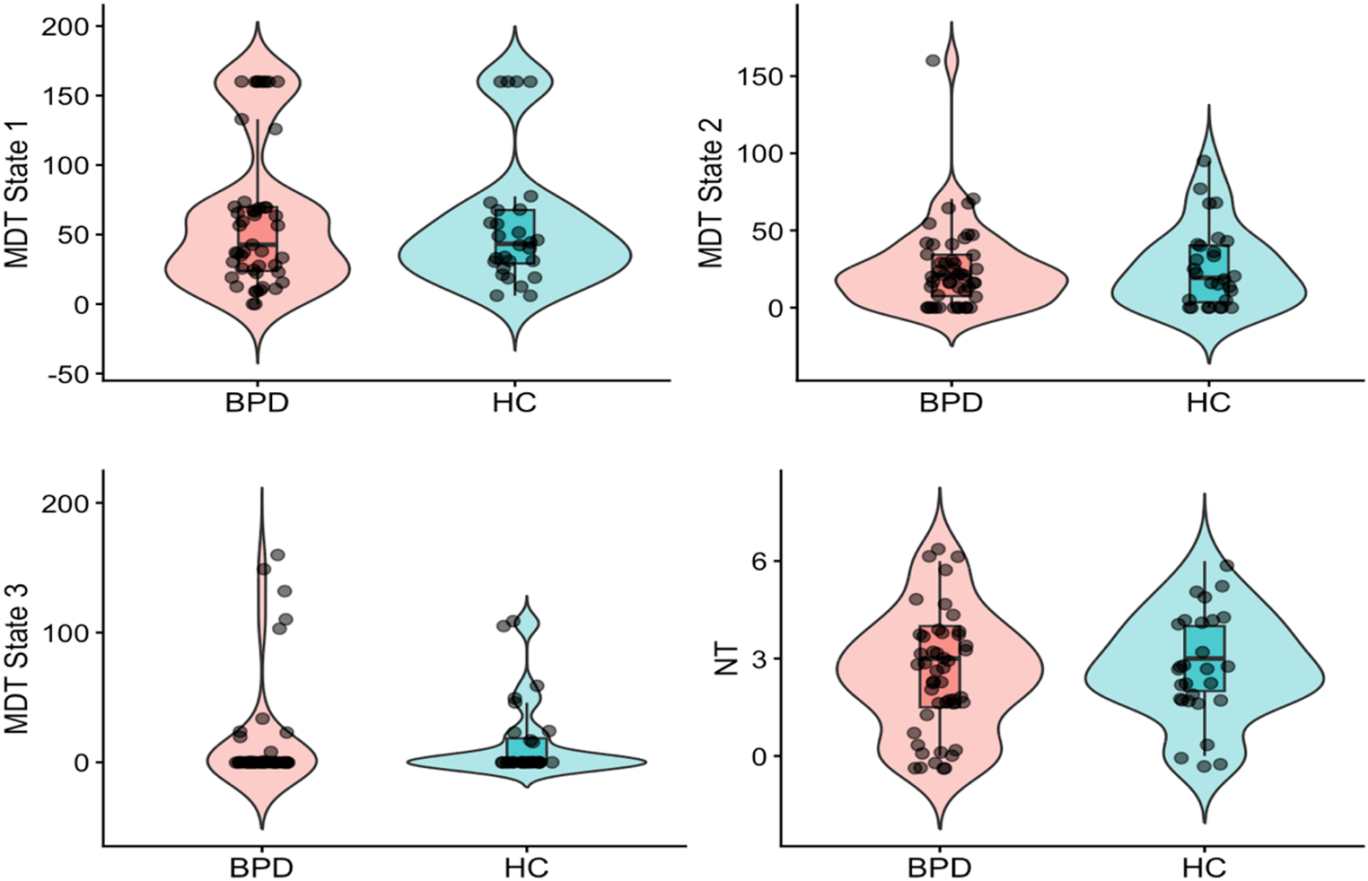
Group Differences in Number of Transitions and Mean Dwell Time spent in Each State. *MDT* Mean dwell time; *NT* Number of transitions

No significant differences were found between the MDT (Fig. 4) in any state for the two groups, or between the NT (Supplementary Material: Table B).

**Fig. 5:**
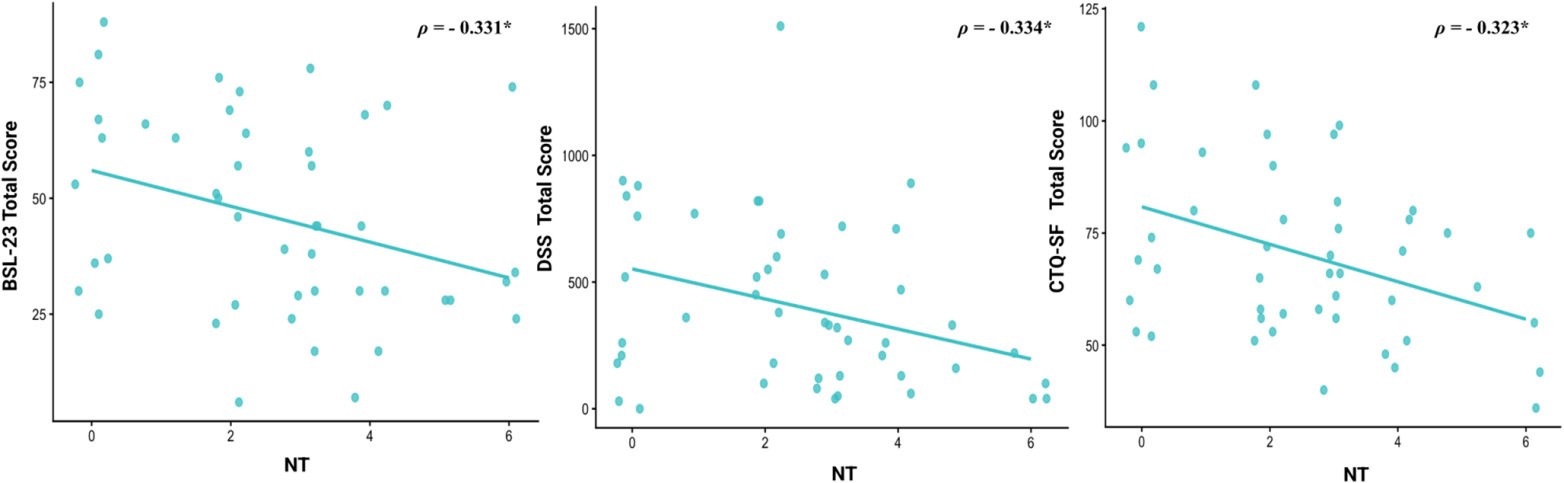
Associations between Number of Transitions and Clinical Symptom Severity in the BPD Group. *ρ* indicates Spearman’s rho. * indicates statistical significance with a *p *< 0.05*. BSL-23* Borderline Symptom List – 23; *DSS* Dissoziations-Spannungs-Skala, *CTQ-SF* Childhood Trauma Questionnaire – Short Form, *NT* Number of transitions

Within-group correlation analyses, however, revealed significant negative correlations between NT and total scores of the BSL-23, *r*(45) = −0.331, *p* = 0.023, total scores of DSS rating scales *r*(45) = −0.334, *p* = 0.022 and total scores of CTQ-SF, *r*(45) = −0.323, *p* = 0.027in the BPD Group (Fig. 5). Correlations of other scales like BIS-11, HAM-D and DERS with the NT did not reach statistical significance (Supplementary Material: Table A).

**Fig. 6:**
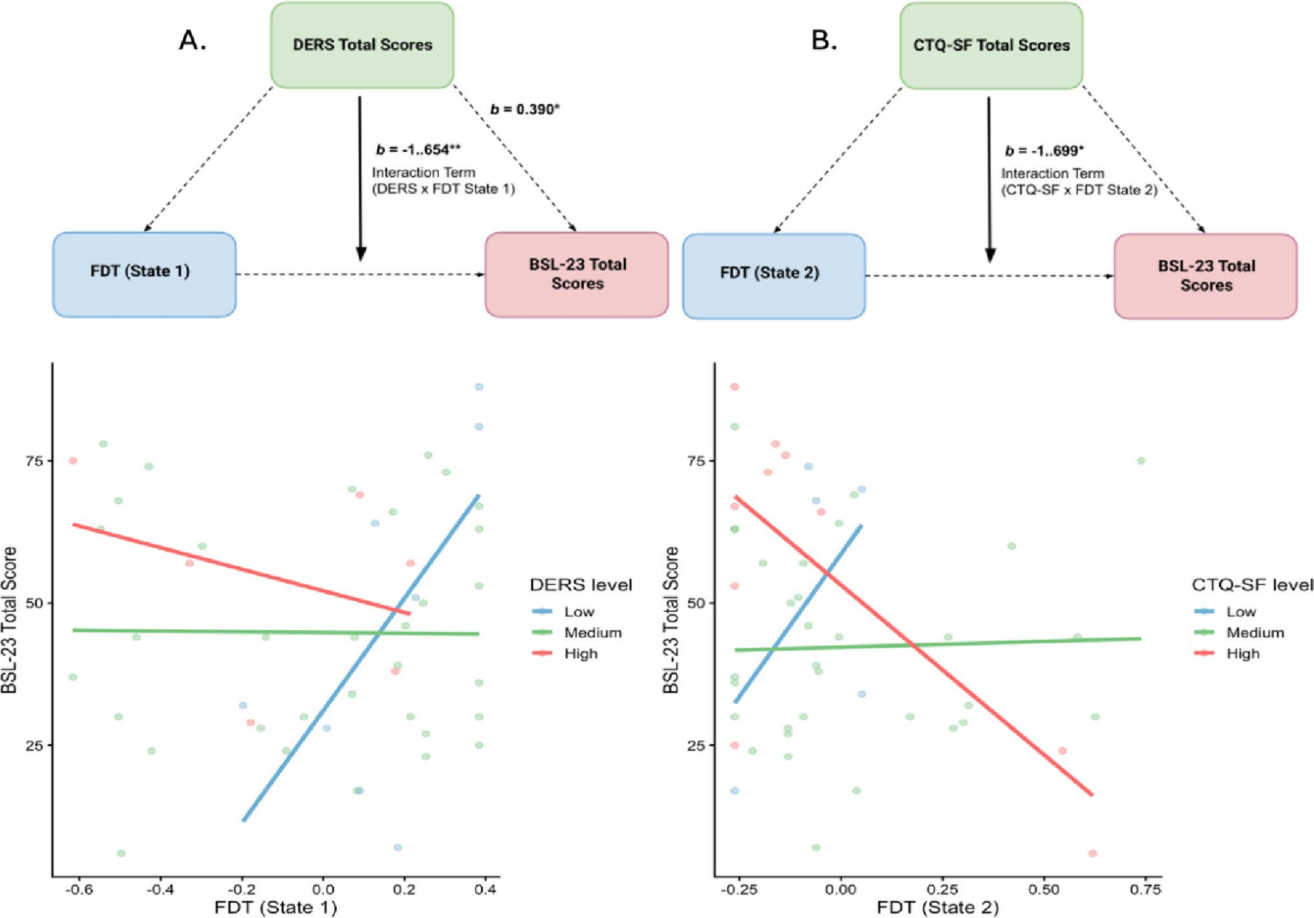
The Moderating Effect of DERS (**A**) and CTQ-SF (**B**) Total Scores on the Association between the BSL-23 scores and FDT spent in State 1 and State 2 by the BPD group respectively. **A** DERS total scores significantly moderate the relationship between BSL-23 scores and FDT spent in State 1. **B** CTQ-SF total scores significantly moderate the relationship between BSL-23 scores and FDT spent in State 2. Lines represent simple slopes at low (−1 SD), medium (mean), and high (+1 SD) moderator levels. Colours blue, green and red indicate low, medium and high scores on (**A**) DERS and (**B**) CTQ-SF respectively. Predictors (FDT State 1 & 2) and Moderators (DERS & CTQ-SF scores) were mean centered for both analyses. *DERS* Difficulty in Emotion Regulation Scale, *CTQ-SF* Childhood Trauma Questionnaire-Short Form

For the moderation analyses, all assumptions necessary for a hierarchical linear regression model were met. Thus, mean centered FDT of states 1 and 2 were taken as predictors in two independent linear regression models with mean centered DERS score as the moderator, in order to reduce multicollinearity. FDT spent in State 3 was not evaluated due to deviation from normality. A significant interaction effect between the DERS total scores and FDT in State 1- but not State 2, on borderline symptom severity was observed (*b* = −1.654, *β* = −0.452, *p =* 0.003) (Fig. 6A). The main effect of DERS scores was also significant (*b* = 0.390, *β* = 0.370, *p =* 0.01). As compared to the base model (Model 0), where DERS scores and FDT spent in State 1 were entered without the interaction term, there was an increase in the explained variance of *R²* change = 0.172 when the interaction term was added to the model (Model 1).

Similarly, the interaction term of CTQ-SF total scores and FDT spent in State 2 was found to significantly predict the total scores of BSL23 (*b* = −1.698, *β* = −0.378, *p =* 0.02). Compared to the base model, where the two predictors were entered without the interaction term, there was an increase in explained variance of *R²* change = 0.096 (Fig. 6B). The main effects of both predictors were not significant. Additionally, the interaction term of CTQ-SF total scores and FDT spent in State 1 was also found to be marginally significant, with a *p =* 0.049. Model specifications are displayed in Tables C1–C4 in the Supplementary Material.

### Robustness checks

Sensitivity analyses were conducted to evaluate the minimum detectable effect sizes given the available sample sizes. For within-group correlation analyses in the BPD sample (*N* = 47), the study had 80% power to detect correlations of approximately |r| ≥ 0.40 at α = 0.05. For between-group comparisons between the BPD (*N* = 47) and HC (*N* = 28) groups, sensitivity analyses indicated that standardized mean differences of approximately d ≥ 0.68 were detectable with 80% power.

Moreover, two separate conditions were explored in the robustness framework for both between-group mean differences as well as within group correlations- (1) Covariate adjustment for age, medication and FD and (2) Exclusion of motion outliers with FD > 0.3 mm(BPD: *n* = 2; HC: *n* = 1) and one BPD participant who reported drowsiness during the fMRI scan, while also controlling for age, medication and FD. For moderation analyses, robust linear regressions were used.

#### Within group analyses

The two conditions, when implemented upon spearman rank correlations between dFNC metrics and clinical scores, revealed distinct results. After controlling for age, medication, and FD, some of the previously observed correlations between dFNC indices and clinical measures remained statistically significant while others did not (Supplementary Material: Table D1). In particular, the association between State Span and DERS scores (*ρ = 0.297*,* p* = 0.050), as well as the association between the NT and DSS scores (ρ *= 0.282*,* p* = 0.060), did not reach statistical significance following covariate adjustment.

While the association between DSS scores and NT remained non-significant across even after exclusion of influential observations (*ρ* = − 0.287, *p* = 0.068)., the relationship between DERS score and State Span re-emerged after exclusion of the drowsy BPD participant and after exclusion of high-motion cases (ρ = 0.357, *p* = 0.022), suggesting partial robustness (Supplementary Material: Table D2). No additional significant associations were observed between dFNC indices and clinical measures. Compared to previous analyses without broader covariate adjustment, stronger correlations between NT and CTQ-SF scores as well as between NT and BSL-23 scores were observed in both sensitivity conditions.

#### Between-group analyses

For between-group analyses, both conditions of robustness checks were implemented using ANCOVAs. In the ANCOVA controlling for age, medication, and FD, no significant main effects of group were found for meta-state measures. Similarly, no significant group effects were observed for cluster-state measures (all *p* > 0.38) (Supplementary Material: Table E1). This pattern remained unchanged after exclusion of motion outliers and the participant reporting drowsiness (all *p* > 0.25; Supplementary Material: Table E2).

#### Moderation analyses

For the moderation model examining the interaction between DERS scores and FDT in State 1 in predicting BSL-23 total scores, the interaction term remained significant in the robust regression model with covariate control (*b* = − 1.650, 95% CI [− 2.635, − 0.665], *p* = 0.002). In this model, the addition of the interaction term resulted in an increase in explained variance compared to the base model (ΔR² = 0.139). The main effect of DERS scores reached statistical significance in the interaction model, whereas the main effect of FDT in State 1 did not (Supplementary Material: Table F1).

Similarly, the moderation model examining the interaction between CTQ-SF scores and FDT in State 2 predicting BSL-23 score, the interaction effect remained statistically significant after robust estimation and covariate adjustment (*b* = − 2.417, 95% CI [− 4.348, − 0.486], *p* = 0.018). Inclusion of the interaction term was associated with an increase in explained variance relative to the base model (Δ*R²* = 0.158; Supplementary Material: Table F2). Main effects of CTQ-SF and FDT in State 2 were not significant.

## Discussion

The aim of this study was to explore dynamic characteristics of functional connectivity in individuals with BPD, using both categorical (BPD vs. HC) and dimensional approaches (within group analyses in the BPD group). While no significant differences between the BPD and HC groups emerged, negative associations between severity of childhood trauma, borderline symptoms and dissociation and NT between states were observed within the BPD group. One positive association was found between ER difficulties and State Span, as estimated using the meta-state approach. Moderation analyses revealed key roles ER difficulties and childhood trauma in influencing the association between borderline symptoms and FDT spent in States 1 and 2 respectively. The descriptive labels assigned to the identified states (Supplementary Material: Table G) facilitate interpretation of these connectivity patterns. Robustness checks conducted under two different sensitivity conditions revealed largely stable findings for associations between dFNC metrics and clinical measures in the BPD group.

Coupled together, these results suggest that dysfunction in brain dynamics, instead of being a categorical feature, tends to manifest dimensionally, more so at the severe end of the BPD continuum, while remaining elusive at less than extreme levels of symptom severity. Similar patterns of progressively unfolding dysfunction in brain dynamics has been reported for a continuum of psychosis spectrum symptoms, showing no to fewer observations of dynamic connectivity alterations in the high risk group but a marked dysfunction in the clinical extreme group [49], as well as for autistic spectrum traits [50], and the attention dysfunction spectrum in neurotypical adolescents [51]. The absence of group differences between BPD and HC groups could, thus, be explained by the heterogeneity of the BPD group which included participants with varying degrees of severity, symptom clusters (e.g. with and without voice hearing), and clinical treatments that are likely associated with different neurobiological profiles. The persistence of null group differences across sensitivity analyses supports this dimensional rather than categorical interpretation of dFNC alterations in BPD. However, the present study was underpowered to detect small group differences and subtle differences in dFNC between BPD and HC groups cannot be ruled out.

The exploration of the relationships between symptom dimensions and dFNC indices in the BPD group obtained using cluster-state and meta-state approaches revealed slightly distinct results. Firstly, using the cluster-state method, the overall findings were in line with previous reports of frequent occurrences in states of weak connectivity (State 1) and less frequent occurrence of states with stronger connectivity in the resting state (States 2 and 3) across all participants [52]. The NT between states was related inversely to BPD symptom load as well as childhood trauma thus supporting the trends of reduced dynamism.

Previous research has described switching patterns between transient brain states not only as markers of mental shifting, complex executive function, subjective cognitive decline, subjective well-being, and cortical arousal 53, 54, 55, 54, 55, 56], but also multiple psychiatric conditions [40, 57, 58]. Moreover, many authors have observed that the tendency to traverse between coordination states may evidence features of metastability [59, 60], defined as the extent of variation in global synchrony, that enables the brain to transition between different states, over time [61, 62]. In a broader sense, metastability is understood to be driven by a well-coordinated balance between integration and segregation tendencies of different brain networks- a characteristic of healthy brain function, without fixating on specific interaction patterns [63, 64]. In this framework, transition dynamics have often been described as potential proxies of brain metastability, by facilitating neural flexibility 65, 66, 66, 67]. Thus, the continuous decrease of NT between states with increasing severity of borderline symptoms may reflect diminishing metastability through patterns of neural ‘fixedness’ driven by misbalanced integration-segregation tendencies [63, 64]. This in turn may manifest as cognitive deficits [68], that may hinder exchange of neural information [69, 70]. Similarly, the negative associations with dissociation and childhood trauma, might be driven by alterations in cortical arousal and information exchange [54] that are facilitated by altered frequency of transitions between brain states, and are often seen in traumatized individuals [71]. In line with these findings, diminished NT has been observed in disorders like schizophrenia [72] and the autism spectrum [73]. Meanwhile, an increased NT in psychiatric samples such as obsessive compulsive disorder [74], have also been reported leading to speculation about the role of unsuccessful inhibition. Thus, similar to past proposals [75, 76], it may be argued that the relationship between state transition dynamics and cognitive functioning may be an inverted U-shaped one [54, 77] where both inadequate as well as excessive NT may mirror dysfunctional neural communication underlying clinical features in diseased populations.

While the NT between states were shown to decline with increasing severity of symptom dimensions, the L1 State Span (i.e. maximum L1 distance covered between two distinct meta-states) as derived using the meta-state approach, displayed a continuous increase with increasing ER difficulties in the BPD group.

This positive correlation could reflect an emotional instability resulting from ER failure, possibly through an ‘inefficient dynamic search process’ [78] giving rise to physiological hyperarousal [79] and erratic shifts in the state space. Furthermore, previous studies lend support to this proposition with heightened emotional reactivity being a key driver of the increased dynamic range in an ADHD cohort [80]. Moreover, increasing spatial variability has been related to unsuccessful ER among neurotypical youth [81]. Similarly, recent rumination correlated with increased dynamic rs-FC between the insula and mPFC among depressed adults [82]. This inverse relationship is mirrored by findings of recent studies underlining the importance of short-range connections through the neural medium in stabilising system dynamics like volatility and metastability [83]. Together, the results of the cluster-state and meta-state correlation analyses show that though individuals with BPD may transition in a limited manner, when they do transition, they tend to navigate an increasingly larger global state space. The pattern of fewer, but long-range transitions between brain states, where brain reconfigurations are infrequent yet impactful suggest diminished dynamic fluidity but an increased dynamic range. This dichotomy complements other contrasting findings of increased BOLD temporal variability in the salience network and reduced variability in the default mode network in a subclinical sample with borderline personality traits [84]. Additionally, research using a graph analysis approach to assess whole-brain functional network found lower global connectivity in patients as well as inverse correlations between characteristic path length and separation insecurity [85]. Together these findings may point towards a more random architecture of neural interactions, thus deviating from the optimal range of small-worldness, giving rise to suboptimal information processing [86] and therefore a more complex relationship of symptom dimensions and brain dynamics in BPD.

Further evidence of the complex and differential role of brain dynamics was found in the significant moderation effect of ER difficulties on the relationship between the FDT spent in a state marked by weak global connectivity (State 1) and borderline symptom severity. The interaction between the FDT spent in State 1 and ER difficulties led to a significant addition in the explained variance of 17%, beyond that of the individual predictors together. This moderation revealed that both very low and very high FDT spent in State 1 could predict either higher or lower BPD symptom severity, depending on the ER capacity. For individuals with better ER abilities, spending less time, proportionally, in State 1 predicted lower BPD severity. This suggests that less time in a weakly connected state, may promote fluid shifts in the state space and greater engagement with a more integrated state, thus enabling access to a broader reservoir of cognitive resources. On the other hand, for those with poorer ER skills, greater FDT spent in State 1 seemed to predict lower BPD severity. This suggests that spending greater proportions of time in a low connectivity state may provide those with poor ER abilities with a compensatory mechanism against ER-related basal hyperarousal in individuals with BPD [79]. Moreover, the temporal stability may help regulate and inhibit volatile and erratic shifts across the state space that accompany ER difficulties, as seen in the positive correlation with State Span.

Analogous findings of the moderating role of childhood trauma were obtained for the relationship between in State 2 and BPD symptom severity, that seems to shift progressively with increasing levels of early-life trauma. While individuals with high childhood trauma tend to report lower borderline severity when they spend larger proportions of time in a well-integrated state, the same tends to have an opposite effect on those with low levels of childhood trauma. These findings are in line with previous reports of a preference for weakly connected states among individuals with PTSD and those with experiences of childhood emotional neglect [57, 87]. Additionally, the combination of childhood trauma and cognitive abilities in BPD samples has been recognized as a significant driver of dissociation [88], a state of consciousness that has commonly been described as a disruption of ‘neuronal and psychological integration’ [89]. Thus, contrary to a state of weak network integration (State 1), spending greater FDT in a state with stronger connectivity (State 2) may reflect a compensatory, possibly cognitive, mechanism against dissociation for BPD patients with high levels of childhood trauma through efficient information integration across networks. On the other hand, for those with lower levels of trauma, spending greater FDT in a state with stronger connectivity at rest may indicate higher inner tension and rigid vigilance [90, 91] that have been reported as basal characteristics of BPD, even after controlling for PTSD or childhood trauma [92].

The interpretation of the findings of this study must follow careful consideration of several potential limitations. First, since the BPD participants were recruited from multiple settings, the patient group was characterized by significant heterogeneity in the levels of severity as well as psychological treatment that the participants were undergoing at the time of assessment. The present findings should be interpreted with consideration of the fact that only female, right-handed participants with BPD were included. This design choice reduced sex-related and lateralization-related variance and enhanced internal validity, but limits generalizability to male patients. Emerging research suggests meaningful sex-linked differences not only in the clinical phenotypes but also in the BPD neurobiology [93]. While male patients tend to embody more externalising symptoms of BPD like impulsivity and aggression, female patients tend to display more internalizing symptoms like self-image issues and affective instability and feelings of emptiness [94]. At the neurobiological level, studies further indicate sex-specific patterns in fronto-limbic circuits, including differential amygdala activation and its connectivity with prefrontal regions (e.g. posterior middle cingulate cortex) during emotion- and aggression-related processing. However, this literature remains limited with few studies directly comparing male and female patients [30]. In light of these preliminary findings, it remains unclear whether the observed dynamic functional connectivity findings would generalize to male.

Thus, the lack of male participants in the current study may limit the application of these findings in clinical practice to some extent. The sample size limited statistical sensitivity for detecting small-to-moderate effects of group mean differences. Sensitivity analyses indicated that the study was powered to detect only moderate-to-large correlations; consequently, null findings should not be interpreted as evidence for the absence of more subtle associations. All analyses were cross-sectional, precluding conclusions about causal relationships between network state transitions, symptom severity, dissociation, and trauma exposure. Additionally, this study relied exclusively on resting-state fMRI, which limits the ability to draw conclusions about neural activity during task-specific cognitive processes. Given the limited prior evidence in this domain, an exploratory approach was appropriate and intended to maximize sensitivity for detecting potentially meaningful patterns. This strategy precluded an a priori analytic plan, which may have limited the specificity of the findings. In this context, statistical analyses were conducted without formal adjustment for multiple comparisons, which increases the risk of type I errors. Accordingly, all results should be interpreted as exploratory and in a hypothesis-generating context. Future studies should aim to replicate these findings in larger, confirmatory samples using appropriate correction procedures, such as false discovery rate (FDR) or permutation-based approaches, to ensure statistical robustness and reproducibility.

The k-means approach to state clustering, despite being a data-driven approach, it is possible that different results may have been obtained using a different number of states. Finally, dFNC has previously been shown to be sensitive to fluctuating arousal states [95], which may have had a confounding effect on the results. Accordingly, post-hoc robustness analyses were conducted to evaluate the effect of such states on key findings, which supported the overall reliability of our findings. With these limitations in mind, the findings of the present study support the notion of wide-ranging associations between neurobiological vulnerability factors and the psychopathology of a borderline personality. Additionally, complex and heterogeneous patterns of intrinsic neural communication that drive and, in some cases, even interact with different symptoms of BPD, to influence disease severity, can be identified using a time varying approach to functional connectivity, highlighting the importance of a functional-domain-dependent approach to research on mental illness in contrast to traditional categorical views. From a translational perspective, emerging neurostimulation approaches in personality disorders may benefit from considering individual differences in brain dynamics, depending on symptom severity, trauma history or levels of emotion dysregulation. While still speculative, the current findings raise the possibility that individual differences in dynamic network organization and symptom severity could be relevant factors to consider in the development of patient-specific stimulation protocols or future studies investigating variability in responses to neuromodulatory interventions.

## Supplementary Information

Below is the link to the electronic supplementary material.

Supplementary Material 1

## Data Availability

The data that support the findings of this study are available from the corresponding author upon reasonable request.
