# Supplementary material for: Dynamic functional connectivity in borderline personality disorder: associations with trauma, emotion regulation and symptom severity
Source: Eur Arch Psychiatry Clin Neurosci. 2026 May 8;276(5):2141–57. doi: 10.1007/s00406-026-02255-5 (PMC13481553; doi:10.1007/s00406-026-02255-5)
Supplement: Supplementary file 1 [file 406_2026_2255_MOESM1_ESM.docx]

**Dynamic Functional Connectivity in Borderline Personality Disorder:**

**Associations with Trauma, Emotion Regulation and Symptom Severity**

**Supplementary Material**

Primary Analyses

**Fig . A.**

*Spearman Rank Correlations between dFNC Indices, Clinical Scores and Quality Control Variables in the BPD Group*

***Note.* C**luster-State dFNC Indices: MDT = Mean Dwell Time. FDT = Fractional Dwell Time. NT = Number of Transitions. BSL-23= Borderline Symptom List -23; HAM-D=Hamilton Depression Scale; BIS-11=Barratt Impulsiveness Scale-11; DSS = Dissoziations-Spannungs-Skala; CTQ-SF=Childhood Trauma Questionnaire-Short Form; DERS=Difficulty in Emotion Regulation Scale. All correlation analyses were two-tailed. RMS = Root Mean Square; FD = Framewise Displacement; Medication = Sum of z-scored Olanzapine and Fluoxetine Dose Equivalents. Warmer Colours represent positive correlations while cooler colours represent negative correlations.

**Fig . B.**

*Spearman Rank Correlations between dFNC Indices and Quality Control Variables including both BPD and HC Groups*

***Note.* C**luster-State dFNC Indices: MDT = Mean Dwell Time. FDT = Fractional Dwell Time. NT = Number of Transitions. All correlation analyses were two-tailed. RMS = Root Mean Square; FD = Framewise Displacement; Medication = Sum of z-scored Olanzapine and Fluoxetine Dose Equivalents. Warmer Colours represent positive correlations while cooler colours represent negative correlations.

**Fig. C**

*Group Differences in Head Motion assessed using Framewise Displacement (left) and Root Mean Square Displacement (right)*

*Note.* Two-tailed independent sample Mann-Whitney *U* tests. FD (left) = Framewise Displacement; RMS (right) = Root Mean Square Displacement.

Table A.

*Spearman Rank Correlations between dFNC indices and clinical scores within the BPD Group*

| **Variable** |  | **HAM-D** | **BSL-23** | **CTQ-SF** | **DERS** | **BIS-11** | **DSS** |
| --- | --- | --- | --- | --- | --- | --- | --- |
| *Meta-State dFNC indices* |  |  |  |  |  |  |  |
| Number of States | Spearman's rho | -0.186 | -0.083 | -0.008 | -0.039 | -0.079 | -0.053 |
|  | p-value | .210 | .578 | .959 | .794 | .600 | .724 |
|  | Lower 95% CI | -0.458 | -0.391 | -0.312 | -0.296 | -0.341 | -0.353 |
|  | Upper 95% CI | 0.119 | 0.260 | 0.307 | 0.234 | 0.210 | 0.260 |
| Changes between States | Spearman's rho | -0.146 | -0.085 | 0.002 | 0.006 | -0.043 | -0.024 |
|  | p-value | .327 | .572 | .991 | .969 | .772 | .875 |
|  | Lower 95% CI | -0.408 | -0.376 | -0.297 | -0.253 | -0.300 | -0.345 |
|  | Upper 95% CI | 0.150 | 0.227 | 0.309 | 0.281 | 0.257 | 0.289 |
| State Span | Spearman's rho | 0.067 | 0.071 | 0.090 | **0.302*** | -0.002 | 0.031 |
|  | p-value | .656 | .635 | .549 | **.039** | .989 | .838 |
|  | Lower 95% CI | -0.222 | -0.231 | -0.222 | **0.038** | -0.321 | -0.254 |
|  | Upper 95% CI | 0.358 | 0.352 | 0.430 | **0.539** | 0.317 | 0.336 |
| Total Distance | Spearman's rho | -0.015 | -0.018 | -0.042 | 0.048 | -0.040 | 0.001 |
|  | p-value | .921 | .903 | .778 | .749 | .790 | .994 |
|  | Lower 95% CI | -0.282 | -0.318 | -0.348 | -0.224 | -0.307 | -0.294 |
|  | Upper 95% CI | 0.266 | 0.278 | 0.271 | 0.320 | 0.246 | 0.312 |
| *Cluster-State dFNC indices* |  |  |  |  |  |  |  |
| MDT State 1 | Spearman's rho | -0.069 | 0.109 | 0.063 | -0.246 | -0.072 | 0.161 |
|  | p-value | .646 | .465 | .676 | .096 | .631 | .279 |
|  | Lower 95% CI | -0.356 | -0.206 | -0.243 | -0.513 | -0.362 | -0.171 |
|  | Upper 95% CI | 0.214 | 0.420 | 0.369 | 0.027 | 0.221 | 0.476 |
| MDT State 2 | Spearman's rho | 0.050 | -0.101 | 0.057 | 0.123 | -0.004 | 0.058 |
|  | p-value | .737 | .500 | .702 | .412 | .978 | .696 |
|  | Lower 95% CI | -0.212 | -0.396 | -0.263 | -0.171 | -0.312 | -0.252 |
|  | Upper 95% CI | 0.344 | 0.196 | 0.380 | 0.391 | 0.282 | 0.368 |
| MDT State 3 | Spearman's rho | 0.095 | 0.062 | -0.113 | 0.105 | -0.046 | -0.134 |
|  | p-value | .526 | .678 | .448 | .481 | .759 | .368 |
|  | Lower 95% CI | -0.191 | -0.232 | -0.391 | -0.209 | -0.343 | -0.419 |
|  | Upper 95% CI | 0.358 | 0.349 | 0.185 | 0.379 | 0.216 | 0.166 |
| NT | Spearman's rho | -0.041 | **-0.331*** | **-0.323*** | -0.095 | -0.084 | **-0.334*** |
|  | p-value | .785 | **.023** | **.027** | .527 | .575 | **.022** |
|  | Lower 95% CI | -0.299 | **-0.592** | **-0.577** | -0.357 | -0.371 | **-0.611** |
|  | Upper 95% CI | 0.217 | **-0.034** | **-0.048** | 0.192 | 0.203 | **-0.016** |
| FDT State 1 | Spearman's rho | -0.075 | 0.069 | 0.013 | -0.277 | -0.051 | 0.136 |
|  | p-value | .617 | .643 | .930 | .059 | .734 | .363 |
|  | Lower 95% CI | -0.353 | -0.244 | -0.291 | -0.523 | -0.328 | -0.181 |
|  | Upper 95% CI | 0.200 | 0.380 | 0.313 | -0.004 | 0.226 | 0.445 |
| FDT State 2 | Spearman's rho | 0.049 | -0.177 | -0.005 | 0.131 | 0.042 | -0.041 |
|  | p-value | .742 | .235 | .975 | .382 | .781 | .785 |
|  | Lower 95% CI | -0.213 | -0.463 | -0.325 | -0.143 | -0.276 | -0.350 |
|  | Upper 95% CI | 0.334 | 0.126 | 0.298 | 0.388 | 0.343 | 0.270 |
| FDT State 3 | Spearman's rho | 0.095 | 0.063 | -0.112 | 0.106 | -0.045 | -0.135 |
|  | p-value | .526 | .675 | .453 | .477 | .763 | .367 |
|  | Lower 95% CI | -0.191 | -0.231 | -0.390 | -0.204 | -0.343 | -0.419 |
|  | Upper 95% CI | 0.358 | 0.349 | 0.185 | 0.379 | 0.216 | 0.166 |

*Note.* 95% CIs based on 1000 bootstrap replicates. * indicates *p* < 0.05. Cluster-state dFNC Indices: MDT = Mean Dwell Time. FDT = Fractional Dwell Time. NT = Number of Transitions. BSL-23= Borderline Symptom List -23; HAM-D=Hamilton Depression Scale; BIS-11=Barratt Impulsiveness Scale-11; DSS=Dissoziations Spannungs Skala; CTQ-SF=Childhood Trauma Questionnaire-Short Form; DERS=Difficulty in Emotion Regulation Scale. All correlation analyses were two-tailed.

| **Table B.**  *Group Differences in dFNC Measures using Independent Samples T-Tests* | | | | | | | |
| --- | --- | --- | --- | --- | --- | --- | --- |
|  | | | | | | **95% CI for Cohen's *d*** | |
|  | ***t*** | ***df*** | ***p*** | **Cohen's *d*** | **SE Cohen's *d*** | **Lower** | **Upper** |
| *Meta-State dFNC indices* |  |  |  |  |  |  |  |
| Number of States | -0.138 | 73 | .890 | -0.033 | 0.239 | -0.501 | 0.435 |
| Changes between States | -0.204 | 73 | .839 | -0.049 | 0.239 | -0.517 | 0.419 |
| State Span | -0.910 | 73 | .366 | -0.217 | 0.240 | -0.686 | 0.253 |
| Total Distance | -1.121 | 73 | .266 | -0.268 | 0.240 | -0.737 | 0.203 |
| *Cluster-State dFNC indices* |  |  |  |  |  |  |  |
| MDT State 1 | 0.478 | 73 | .634 | 0.114 | 0.239 | -0.354 | 0.582 |
| MDT State 2 | -0.098 | 73 | .922 | -0.023 | 0.239 | -0.491 | 0.445 |
| MDT State 3 | 0.025 | 73 | .980 | 0.006 | 0.239 | -0.462 | 0.474 |
| NT | -0.527 | 73 | .599 | -0.126 | 0.239 | -0.594 | 0.343 |
| FDT State 1 | 0.342 | 73 | .733 | 0.082 | 0.239 | -0.387 | 0.550 |
| FDT State 2 | -0.162 | 73 | .872 | -0.039 | 0.239 | -0.506 | 0.429 |
| FDT State 3 | -0.250 | 73 | .804 | -0.060 | 0.239 | -0.527 | 0.409 |
|  | | | | | | | |
| Note.  Two -Tailed T-Tests. Cluster-State dFNC Indices: MDT = Mean Dwell Time. FDT = Fractional Dwell Time. NT = Number of Transitions | | | | | | | |

**Table C.1**

*Moderation Analysis of the Role of Emotion Regulation Difficulties in the Association between Fractional Dwell Time in State 1 and Borderline Symptom Severity*

| *Model Summary – BSL-23 Total Score* | | | | | | | | | | | | | | | | | | |
| --- | --- | --- | --- | --- | --- | --- | --- | --- | --- | --- | --- | --- | --- | --- | --- | --- | --- | --- |
| **Model** | | ***R*** | | ***R²*** | | **Adjusted *R²*** | | **RMSE** | | ***R²* Change** | | ***df_1_*** | | ***df_2_*** | | ***p*** | | |
| M₀ |  | 0.261 |  | 0.068 |  | 0.026 |  | 21.007 |  | 0.068 |  | 2 |  | 44 |  | 0.213 |  |  |
| M₁ |  | 0.490 |  | 0.240 |  | 0.187 |  | 19.189 |  | 0.172 |  | 1 |  | 43 |  | 0.003 | * |  |
|  | | | | | | | | | | | | | | | | | | |

| *ANOVA* | | | | | | | | | | | | | | |
| --- | --- | --- | --- | --- | --- | --- | --- | --- | --- | --- | --- | --- | --- | --- |
| **Model** | |  | | **Sum of Squares** | | ***df*** | | **Mean Square** | | ***F*** | | ***p*** | | |
| M₀ |  | Regression |  | 1416.077 |  | 2 |  | 708.039 |  | 1.604 |  | 0.213 |  |  |
|  |  | Residual |  | 19417.795 |  | 44 |  | 441.314 |  |  |  |  |  |  |
|  |  | Total |  | 20833.872 |  | 46 |  |  |  |  |  |  |  |  |
| M₁ |  | Regression |  | 5000.944 |  | 3 |  | 1666.981 |  | 4.527 |  | 0.008 | * |  |
|  |  | Residual |  | 15832.928 |  | 43 |  | 368.208 |  |  |  |  |  |  |
|  |  | Total |  | 20833.872 |  | 46 |  |  |  |  |  |  |  |  |
|  | | | | | | | | | | | | | | |

| *Coefficients* | | | | | | | | |
| --- | --- | --- | --- | --- | --- | --- | --- | --- |
|  | | | | | | | **95% CI** | |
| **Model** |  | **Unstandardized** | **Standard Error** | **Standardized** | ***t*** | ***p*** | **Lower** | **Upper** |
| M₀ | (Intercept) | 46.213 | 3.064 |  | 15.081 | < .001* | 40.037 | 52.388 |
|  | FDT State 1 | 7.264 | 9.985 | 0.111 | 0.727 | .471 | -12.861 | 27.388 |
|  | DERS | 0.288 | 0.161 | 0.273 | 1.782 | .082 | -0.038 | 0.613 |
| M₁ | (Intercept) | 42.907 | 2.993 |  | 14.337 | < .001* | 36.871 | 48.942 |
|  | FDT State 1 | 19.283 | 9.901 | 0.296 | 1.948 | .058 | -0.684 | 39.251 |
|  | DERS | 0.390 | 0.151 | 0.370 | 2.581 | .013* | 0.085 | 0.694 |
|  | FDT State 1  ✻   DERS | -1.654 | 0.530 | -0.452 | -3.120 | .003* | -2.723 | -0.585 |
| *Note.*  M₁ =mean centered FDT State 1, mean centered DERS, mean centered FDT State 1 : mean centered DERS | | | | | | | | |

*Note.* Hierarchical Linear Regression Model was used to assess the interaction between FDT State 1 and DERS Scores.

FDT State 1 values and DERS Scores were entered after mean centering. * incidcates *p* < 0.05.

M₀ = mean centered FDT State 1, mean centered DERS, M₁ = mean centered FDT State 1, mean centered DERS, mean centered FDT State 1 : mean centered DERS. DERS = Difficulty in Emotion Regulation Scale. FDT = Fractional Dwell Time

**Table C.2**

*Moderation Analysis of the Role of Emotion Regulation Difficulties in the Association between Fractional Dwell Time in State 2 and Borderline Symptom Severity*

| *Model Summary - BSL23.Total.Score* | | | | | | | | | | | | | | | | | | | |
| --- | --- | --- | --- | --- | --- | --- | --- | --- | --- | --- | --- | --- | --- | --- | --- | --- | --- | --- | --- |
| **Model** | | ***R*** | | ***R²*** | | **Adjusted *R²*** | | **RMSE** | | ***R²* Change** | | ***df1*** | | ***df2*** | | ***p*** | | |  |
| M₀ |  | 0.355 |  | 0.126 |  | 0.086 |  | 20.346 |  | 0.126 |  | 2 |  | 44 |  | 0.052 |  |  |  |
| M₁ |  | 0.419 |  | 0.175 |  | 0.118 |  | 19.987 |  | 0.050 |  | 1 |  | 43 |  | 0.114 |  |  |  |

| *ANOVA* | | | | | | | | | | | | | | |
| --- | --- | --- | --- | --- | --- | --- | --- | --- | --- | --- | --- | --- | --- | --- |
| **Model** | |  | | **Sum of Squares** | | ***df*** | | **Mean Square** | | ***F*** | | ***p*** | | |
| M₀ |  | Regression |  | 2618.761 |  | 2 |  | 1309.380 |  | 3.163 |  | 0.052 |  |  |
|  |  | Residual |  | 18215.112 |  | 44 |  | 413.980 |  |  |  |  |  |  |
|  |  | Total |  | 20833.872 |  | 46 |  |  |  |  |  |  |  |  |
| M₁ |  | Regression |  | 3655.940 |  | 3 |  | 1218.647 |  | 3.051 |  | 0.039 | * |  |
|  |  | Residual |  | 17177.932 |  | 43 |  | 399.487 |  |  |  |  |  |  |
|  |  | Total |  | 20833.872 |  | 46 |  |  |  |  |  |  |  |  |

| *Coefficients* | | | | | | | | |
| --- | --- | --- | --- | --- | --- | --- | --- | --- |
|  | | | | | | | **95% CI** | |
| **Model** |  | **Unstandardized** | **Standard Error** | **Standardized** | ***t*** | ***p*** | **Lower** | **Upper** |
| M₀ | (Intercept) | 46.213 | 2.968 |  | 15.571 | < .001 | 40.231 | 52.194 |
|  | DERS | 0.304 | 0.151 | 0.289 | 2.011 | .051 | -0.000 | 0.609 |
|  | FDT State 2 | -20.502 | 11.007 | -0.267 | -1.863 | .069 | -42.69 | 1.682 |
| M₁ | (Intercept) | 45.292 | 2.971 |  | 15.246 | < .001 | 39.301 | 51.284 |
|  | DERS | 0.311 | 0.149 | 0.295 | 2.091 | .042* | 0.011 | 0.611 |
|  | FDT State 2 | -26.009 | 11.340 | -0.339 | -2.294 | .027* | -48.88 | -3.139 |
|  | DERS  ✻   FDT State 2 | 0.891 | 0.553 | 0.234 | 1.611 | .114 | -0.224 | 2.006 |
|  | | | | | | | | |

*Note.* Hierarchical Linear Regression Model was used to test the interaction between FDT State 2 and DERS Scores. FDT State 2 values and DERS Scores were entered after mean centering. * indicates *p* < 0.05. M₀ = mean centered FDT State 2, mean centered DERS, M₁ = mean centered FDT State 2, mean centered DERS, mean centered FDT State 2 : mean centered DERS

DERS = Difficulty in Emotion Regulation Scale. FDT = Fractional Dwell Time

**Table C.3**

*Moderation Analysis of the Role of Childhood Trauma in the Association between Fractional Dwell Time in State 1 and Borderline Symptom Severity*

*Model Summary - BSL23.Total.Score*

| **Model** | ***R*** | ***R²*** | **Adjusted *R²*** | **RMSE** | ***R²* Change** | ***df_1_*** | ***df_2_*** | ***p*** |
| --- | --- | --- | --- | --- | --- | --- | --- | --- |
| M₀ | 0.208 | 0.043 | -0.000 | 21.285 | 0.043 | 2 | 44 | .379 |
| M₁ | 0.356 | 0.127 | 0.066 | 20.571 | 0.083 | 1 | 43 | .049 |

| *ANOVA* | | | | | | |
| --- | --- | --- | --- | --- | --- | --- |
| **Model** |  | **Sum of Squares** | ***df*** | **Mean Square** | ***F*** | ***p*** |
| M₀ | Regression | 899.109 | 2 | 449.555 | 0.992 | .379 |
|  | Residual | 19934.763 | 44 | 453.063 |  |  |
|  | Total | 20833.872 | 46 |  |  |  |
| M₁ | Regression | 2636.896 | 3 | 878.965 | 2.077 | .117 |
|  | Residual | 18196.976 | 43 | 423.185 |  |  |
|  | Total | 20833.872 | 46 |  |  |  |
|  | | | | | | |

| *Coefficients* | | | | | | | | |
| --- | --- | --- | --- | --- | --- | --- | --- | --- |
|  | | | | | | | **95% CI** | |
| **Model** |  | **Unstandardized** | **Standard Error** | **Standardized** | ***t*** | ***p*** | **Lower** | **Upper** |
| M₀ | (Intercept) | 46.213 | 3.105 |  | 14.884 | < .001 | 39.956 | 52.470 |
|  | CTQ-SF | 0.225 | 0.161 | 0.206 | 1.397 | .169 | -0.100 | 0.550 |
|  | FDT State 1 | 1.472 | 9.621 | 0.023 | 0.153 | .879 | -17.917 | 20.861 |
| M₁ | (Intercept) | 46.083 | 3.001 |  | 15.354 | < .001 | 40.030 | 52.136 |
|  | CTQ-SF | 0.124 | 0.164 | 0.114 | 0.760 | .451 | -0.206 | 0.454 |
|  | FDT State 1 | -3.642 | 9.634 | -0.056 | -0.378 | .707 | -23.072 | 15.788 |
|  | CTQ-SF  ✻   FDT State 1 | 1.026 | 0.506 | 0.314 | 2.026 | .049 | 0.005 | 2.047 |
|  | | | | | | | | |

Note. Hierarchical Linear Regression Model was used to assess the interaction between FDT State 1 and CTQ-SF Scores. FDT State 1 values and CTQ-SF Scores were entered after mean centering.

 M₀ = mean centered FDT State 1 , mean centered CTQ-SF

M₁ = mean centered FDT State 1 , mean centered CTQ-SF, mean centered FDT State 1: mean centered CTQ-SF

CTQ-SF = Childhood Trauma Questionnaire-Short Form. FDT = Fractional Dwell Time.

## Table C.4

## *Moderation Analysis of the Role of Childhood Trauma in the Association between Fractional Dwell Time in State 2 and Borderline Symptom Severity*

| *Model Summary - BSL23.Total.Score* | | | | | | | | |
| --- | --- | --- | --- | --- | --- | --- | --- | --- |
| **Model** | ***R*** | ***R²*** | **Adjusted *R²*** | **RMSE** | ***R²* Change** | ***df_1_*** | ***df_2_*** | ***p*** |
| M₀ | 0.307 | 0.094 | 0.053 | 20.711 | 0.094 | 2 | 44 | .114 |
| M₁ | 0.437 | 0.191 | 0.134 | 19.804 | 0.096 | 1 | 43 | .029* |
|  | | | | | | | | |

| *ANOVA* | | | | | | |
| --- | --- | --- | --- | --- | --- | --- |
| **Model** |  | **Sum of Squares** | ***df*** | **Mean Square** | ***F*** | ***p*** |
| M₀ | Regression | 1959.581 | 2 | 979.791 | 2.284 | .114 |
|  | Residual | 18874.291 | 44 | 428.961 |  |  |
|  | Total | 20833.872 | 46 |  |  |  |
| M₁ | Regression | 3969.827 | 3 | 1323.276 | 3.374 | .027* |
|  | Residual | 16864.046 | 43 | 392.187 |  |  |
|  | Total | 20833.872 | 46 |  |  |  |
|  | | | | | | |

| *Coefficients* | | | | | | | | |
| --- | --- | --- | --- | --- | --- | --- | --- | --- |
|  | | | | | | | **95% CI** | |
| **Model** |  | **Unstandardized** | **Standard Error** | **Standardized** | ***t*** | ***p*** | **Lower** | **Upper** |
| M₀ | (Intercept) | 46.213 | 3.021 |  | 15.297 | < .001* | 40.124 | 52.301 |
|  | CTQ-SF | 0.242 | 0.157 | 0.221 | 1.538 | .131 | -0.075 | 0.559 |
|  | FDT State 2 | -17.424 | 11.027 | -0.227 | -1.580 | .121 | -39.647 | 4.799 |
| M₁ | (Intercept) | 46.791 | 2.900 |  | 16.135 | < .001* | 40.943 | 52.639 |
|  | CTQ-SF | 0.072 | 0.168 | 0.066 | 0.431 | .668 | -0.266 | 0.411 |
|  | FDT State 2 | -5.137 | 11.858 | -0.067 | -0.433 | .667 | -29.052 | 18.778 |
|  | CTQ-SF  ✻   FDT State 2 | -1.699 | 0.750 | -0.378 | -2.264 | .029* | -3.212 | -0.186 |
|  | | | | | | | | |

| Note. Hierarchical Linear Regression Model was used to assess the interaction between FDT State 2 and CTQ-SF Scores.  FDT State 2 values and CTQ-SF Scores were entered after mean centering. * indicates *p* < 0.05.  M₀ = mean centered FDT State 2 , mean centered CTQ-SF  M_1_ = mean centered FDT State 2 , mean centered CTQ-SF, mean centered FDT State 2 : mean centered CTQ-SF  CTQ-SF = Childhood Trauma Questionnaire-Short Form. FDT = Fractional Dwell Time. |  |
| --- | --- |

**Supplementary Material**

**Robustness Checks**

**Table D.1.**

*Spearman Partial Correlations between Clinical Scores and dFNC Metrics of the BPD Group after controlling for Age, Medication and Framewise Displacement*

| **Variable** |  | **BSL-23** | **HAM-D** | **CTQ-SF** | **DERS** | **BIS-11** | **DSS** |
| --- | --- | --- | --- | --- | --- | --- | --- |
| *Meta-State dFNC indices* |  |  |  |  |  |  |  |
| Number of States | Spearman's rho | -0.098 | -0.176 | -0.050 | -0.061 | -0.099 | -0.034 |
|  | p-value | .525 | .254 | .749 | .692 | .521 | .828 |
|  | Lower 95% CI | -0.440 | -0.447 | -0.380 | -0.353 | -0.372 | -0.401 |
|  | Upper 95% CI | 0.270 | 0.139 | 0.305 | 0.260 | 0.230 | 0.315 |
| Changes between States | Spearman's rho | -0.092 | -0.141 | -0.021 | 0.004 | -0.045 | -0.014 |
|  | p-value | .554 | .361 | .892 | .980 | .770 | .931 |
|  | Lower 95% CI | -0.401 | -0.408 | -0.350 | -0.291 | -0.328 | -0.383 |
|  | Upper 95% CI | 0.249 | 0.148 | 0.310 | 0.314 | 0.278 | 0.337 |
| State Span | Spearman's rho | 0.062 | 0.089 | 0.054 | 0.297 | -0.025 | 0.065 |
|  | p-value | .690 | .567 | .728 | .050 | .871 | .676 |
|  | Lower 95% CI | -0.245 | -0.240 | -0.297 | -0.023 | -0.360 | -0.262 |
|  | Upper 95% CI | 0.367 | 0.391 | 0.407 | 0.562 | 0.325 | 0.396 |
| Total Distance | Spearman's rho | -0.025 | -0.009 | -0.068 | 0.047 | -0.042 | 0.011 |
|  | p-value | .871 | .956 | .661 | .760 | .785 | .943 |
|  | Lower 95% CI | -0.360 | -0.280 | -0.394 | -0.262 | -0.330 | -0.341 |
|  | Upper 95% CI | 0.319 | 0.273 | 0.282 | 0.340 | 0.271 | 0.347 |
| *Cluster-State dFNC indices* |  |  |  |  |  |  |  |
| MDT State 1 | Spearman's rho | 0.143 | -0.086 | 0.135 | -0.225 | -0.042 | 0.160 |
|  | p-value | .356 | .577 | .383 | .142 | .785 | .300 |
|  | Lower 95% CI | -0.224 | -0.388 | -0.229 | -0.509 | -0.359 | -0.163 |
|  | Upper 95% CI | 0.450 | 0.228 | 0.445 | 0.067 | 0.271 | 0.470 |
| MDT State 2 | Spearman's rho | -0.117 | 0.072 | 0.013 | 0.112 | -0.019 | 0.095 |
|  | p-value | .449 | .643 | .934 | .470 | .904 | .541 |
|  | Lower 95% CI | -0.407 | -0.220 | -0.303 | -0.205 | -0.364 | -0.181 |
|  | Upper 95% CI | 0.227 | 0.399 | 0.361 | 0.434 | 0.312 | 0.390 |
| MDT State 3 | Spearman's rho | 0.050 | 0.125 | -0.186 | 0.096 | -0.061 | -0.101 |
|  | p-value | .747 | .420 | .228 | .534 | .696 | .512 |
|  | Lower 95% CI | -0.258 | -0.201 | -0.481 | -0.225 | -0.341 | -0.412 |
|  | Upper 95% CI | 0.346 | 0.415 | 0.151 | 0.378 | 0.254 | 0.211 |
| NT | Spearman's rho | **-0.356*** | -6.115×10^-4^ | **-0.409**** | -0.110 | -0.089 | -0.282 |
|  | p-value | **.018** | .997 | **.006** | .476 | .567 | .063 |
|  | Lower 95% CI | **-0.624** | -0.308 | **-0.638** | -0.377 | -0.382 | -0.604 |
|  | Upper 95% CI | **-0.051** | 0.313 | **-0.114** | 0.188 | 0.222 | 0.007 |
| FDT State 1 | Spearman's rho | 0.096 | -0.099 | 0.078 | -0.260 | -0.019 | 0.118 |
|  | p-value | .534 | .521 | .617 | .089 | .900 | .445 |
|  | Lower 95% CI | -0.268 | -0.412 | -0.279 | -0.523 | -0.337 | -0.203 |
|  | Upper 95% CI | 0.416 | 0.213 | 0.399 | 0.037 | 0.284 | 0.429 |
| FDT State 2 | Spearman's rho | -0.188 | 0.065 | -0.027 | 0.113 | 0.021 | -0.019 |
|  | p-value | .222 | .673 | .860 | .465 | .894 | .905 |
|  | Lower 95% CI | -0.474 | -0.232 | -0.327 | -0.189 | -0.314 | -0.324 |
|  | Upper 95% CI | 0.152 | 0.364 | 0.321 | 0.422 | 0.349 | 0.271 |
| FDT State 3 | Spearman's rho | 0.050 | 0.125 | -0.186 | 0.096 | -0.061 | -0.102 |
|  | p-value | .747 | .418 | .228 | .534 | .694 | .511 |
|  | Lower 95% CI | -0.257 | -0.200 | -0.481 | -0.223 | -0.343 | -0.413 |
|  | Upper 95% CI | 0.346 | 0.415 | 0.152 | 0.378 | 0.252 | 0.211 |

*Note*. 95% CI were extracted from 1000 bootstraps. * indicates p < 0.05. MDT = Mean Dwell Time. FDT = Fractional Dwell Time. NT = Number of Transitions. BSL-23= Borderline Symptom List -23; HAM-D=Hamilton Depression Scale; BIS-11=Barratt Impulsiveness Scale-11; DSS = Dissoziations-Spannungs-Skala; CTQ-SF=Childhood Trauma Questionnaire-Short Form; DERS=Difficulty in Emotion Regulation Scale. All correlation analyses were two-tailed.

**Table D.2**

*Spearman Partial Correlations between Clinical Scores and dFNC Metrics of the BPD Group after Covariate Control (Age, Medication, Framewise Displacement) and Exclusion of Influential Points*

| **Variable** |  | **HAM-D** | **BSL-23** | **CTQ-SF** | **DERS** | **BIS-11** | **DSS** |
| --- | --- | --- | --- | --- | --- | --- | --- |
| *Meta-State dFNC indices* |  |  |  |  |  |  |  |
| Number of States | Spearman's rho | -0.155 | -0.067 | -0.080 | -0.050 | -0.083 | -0.001 |
|  | p-value | .333 | .677 | .621 | .755 | .606 | .993 |
|  | Lower 95% CI | -0.430 | -0.418 | -0.430 | -0.364 | -0.366 | -0.371 |
|  | Upper 95% CI | 0.187 | 0.319 | 0.288 | 0.287 | 0.240 | 0.367 |
| Changes between States | Spearman's rho | -0.117 | -0.062 | -0.028 | 0.006 | -0.036 | 0.038 |
|  | p-value | .465 | .698 | .864 | .971 | .824 | .815 |
|  | Lower 95% CI | -0.381 | -0.379 | -0.366 | -0.304 | -0.317 | -0.323 |
|  | Upper 95% CI | 0.213 | 0.319 | 0.328 | 0.322 | 0.267 | 0.388 |
| State Span | Spearman's rho | 0.151 | 0.153 | 0.000 | **0.357*** | 0.013 | 0.134 |
|  | p-value | .345 | .341 | .995 | **.022** | .935 | .404 |
|  | Lower 95% CI | -0.164 | -0.155 | -0.361 | **0.044** | -0.342 | -0.204 |
|  | Upper 95% CI | 0.458 | 0.472 | 0.366 | **0.640** | 0.360 | 0.434 |
| Total Distance | Spearman's rho | 0.023 | -0.009 | -0.043 | 0.048 | -0.065 | 0.071 |
|  | p-value | .885 | .956 | .792 | .766 | .688 | .660 |
|  | Lower 95% CI | -0.244 | -0.321 | -0.391 | -0.266 | -0.346 | -0.278 |
|  | Upper 95% CI | 0.340 | 0.355 | 0.313 | 0.367 | 0.233 | 0.382 |
| *Cluster-State dFNC indices* |  |  |  |  |  |  |  |
| MDT State 1 | Spearman's rho | -0.141 | 0.150 | 0.065 | -0.226 | 0.028 | 0.090 |
|  | p-value | .380 | .348 | .688 | .155 | .863 | .578 |
|  | Lower 95% CI | -0.456 | -0.242 | -0.325 | -0.482 | -0.290 | -0.256 |
|  | Upper 95% CI | 0.200 | 0.478 | 0.424 | 0.073 | 0.342 | 0.432 |
| MDT State 2 | Spearman's rho | 0.031 | -0.128 | 0.066 | 0.159 | -0.120 | 0.127 |
|  | p-value | .847 | .426 | .681 | .322 | .455 | .429 |
|  | Lower 95% CI | -0.266 | -0.434 | -0.277 | -0.170 | -0.451 | -0.188 |
|  | Upper 95% CI | 0.363 | 0.234 | 0.413 | 0.476 | 0.202 | 0.422 |
| MDT State 3 | Spearman's rho | 0.221 | 0.038 | -0.142 | 0.033 | -0.100 | -0.035 |
|  | p-value | .164 | .811 | .376 | .836 | .534 | .828 |
|  | Lower 95% CI | -0.093 | -0.285 | -0.445 | -0.301 | -0.403 | -0.373 |
|  | Upper 95% CI | 0.504 | 0.331 | 0.201 | 0.377 | 0.229 | 0.299 |
| NT | Spearman's rho | -0.043 | **-0.424*** | **-0.369*** | -0.116 | -0.217 | -0.287 |
|  | p-value | .792 | **.006** | **.018** | .468 | .173 | .068 |
|  | Lower 95% CI | -0.332 | **-0.688** | **-0.606** | -0.423 | -0.513 | -0.595 |
|  | Upper 95% CI | 0.264 | **-0.116** | **-0.041** | 0.191 | 0.089 | 0.043 |
| FDT State 1 | Spearman's rho | -0.154 | 0.090 | 0.024 | -0.263 | 0.035 | 0.046 |
|  | p-value | .338 | .574 | .883 | .097 | .829 | .773 |
|  | Lower 95% CI | -0.455 | -0.302 | -0.352 | -0.518 | -0.281 | -0.310 |
|  | Upper 95% CI | 0.190 | 0.428 | 0.383 | 0.040 | 0.355 | 0.386 |
| FDT State 2 | Spearman's rho | 0.030 | -0.192 | 0.000 | 0.166 | -0.053 | -0.009 |
|  | p-value | .851 | .229 | .995 | .298 | .744 | .958 |
|  | Lower 95% CI | -0.255 | -0.477 | -0.309 | -0.144 | -0.392 | -0.301 |
|  | Upper 95% CI | 0.351 | 0.172 | 0.357 | 0.465 | 0.258 | 0.301 |
| FDT State 3 | Spearman's rho | 0.222 | 0.039 | -0.142 | 0.034 | -0.101 | -0.035 |
|  | p-value | .163 | .811 | .375 | .835 | .530 | .828 |
|  | Lower 95% CI | -0.092 | -0.285 | -0.447 | -0.300 | -0.402 | -0.375 |
|  | Upper 95% CI | 0.505 | 0.331 | 0.201 | 0.377 | 0.229 | 0.299 |

*Note.* One participant reporting short-term drowsiness during the fMRI Scan as well as two participants with FD > 0.3mm were excluded from these analyses. 95% CI were extracted from 1000 bootstraps. * indicates p <0.05. Cluster-State dFNC Indices: MDT = Mean Dwell Time. FDT = Fractional Dwell Time. NT = Number of Transitions. BSL-23= Borderline Symptom List -23; HAM-D=Hamilton Depression Scale; BIS-11=Barratt Impulsiveness Scale-11; DSS = Dissoziations-Spannungs-Skala; CTQ-SF=Childhood Trauma Questionnaire-Short Form; DERS=Difficulty in Emotion Regulation Scale; FD = Framewise Displacement. All correlation analyses were two-tailed.

**Table E.1.**

*ANCOVA results for dynamic functional connectivity metrics after controlling for Age, Medication and Framewise Displacement*

| **Dependent variable** | ***F(df₁, df₂)*** | ***p*** | ***η²_p_*** | **Lower 95% CI** | **Upper 95% CI** |
| --- | --- | --- | --- | --- | --- |
| *Meta-State dFNC indices* |  |  |  |  |  |
| Number of States | 0.01 (1, 70) | 0.913 | 0.000 | 0.000 | 0.035 |
| Changes Between States | 0.03 (1, 70) | 0.857 | 0.000 | 0.000 | 0.048 |
| State Span | 0.09 (1, 70) | 0.770 | 0.001 | 0.000 | 0.061 |
| Total Distance | 0.76 (1, 70) | 0.386 | 0.011 | 0.000 | 0.103 |
| *Cluster-State dFNC indices* |  |  |  |  |  |
| MDT State 1 | 0.00 (1, 70) | 0.970 | 0.000 | 0.000 | 0.000 |
| MDT State 2 | 0.12 (1, 70) | 0.734 | 0.002 | 0.000 | 0.066 |
| MDT State 3 | 0.28 (1, 70) | 0.598 | 0.004 | 0.000 | 0.080 |
| NT | 0.02 (1, 70) | 0.883 | 0.000 | 0.000 | 0.042 |
| FDT State 1 | 0.00 (1, 70) | 0.945 | 0.000 | 0.000 | 0.000 |
| FDT State 2 | 0.11 (1, 70) | 0.744 | 0.002 | 0.000 | 0.064 |
| FDT State 3 | 0.07 (1, 70) | 0.795 | 0.000 | 0.000 | 0.058 |

*Note*. ANCOVAs compared BPD and healthy control groups while controlling for age, framewise displacement, and medication. Effect sizes are reported as partial eta squared (*η²_p_*). Cluster-State dFNC Indices: MDT = Mean Dwell Time. FDT = Fractional Dwell Time. NT = Number of Transitions. All analyses were two-tailed.

**Table E.2**

*ANCOVA results for dynamic functional connectivity metrics after exclusion of influential points and controlling for Age, Medication and Framewise Displacement*

| **Dependent Variable** | ***F(df₁, df₂)*** | ***p*** | ***η²_p_*** | **95% CI**  **Lower** | **95% CI**  **Upper** |
| --- | --- | --- | --- | --- | --- |
| *Meta-State dFNC indices* |  |  |  |  |  |
| Number of States | 0.00 (1, 66) | 0.950 | 0.000 | 0.000 | 0.000 |
| Changes Between States | 0.19 (1, 66) | 0.664 | 0.003 | 0.000 | 0.077 |
| State Span | 0.30 (1, 66) | 0.587 | 0.004 | 0.000 | 0.086 |
| Total Distance | 1.32 (1, 66) | 0.254 | 0.020 | 0.000 | 0.128 |
| *Cluster-State dFNC indices* |  |  |  |  |  |
| MDT State 1 | 0.07 (1, 66) | 0.789 | 0.001 | 0.000 | 0.062 |
| MDT State 2 | 0.13 (1, 66) | 0.716 | 0.002 | 0.000 | 0.072 |
| MDT State 3 | 0.00 (1, 66) | 0.965 | 0.000 | 0.000 | 0.000 |
| NT | 0.00 (1, 66) | 0.956 | 0.000 | 0.000 | 0.000 |
| FDT State 1 | 0.29 (1, 66) | 0.591 | 0.004 | 0.000 | 0.085 |
| FDT State 2 | 0.11 (1, 66) | 0.738 | 0.002 | 0.000 | 0.069 |
| FDT State 3 | 0.10 (1, 66) | 0.756 | 0.001 | 0.000 | 0.067 |

*Note*. ANCOVAs compared BPD and healthy control groups while controlling for age, framewise displacement, and medication. Effect sizes are reported as partial eta squared (*η²_p_*). One BPD participant reporting short-term drowsiness during the fMRI Scan, two BPD participants and one HC participant with framewise displacement (FD) > 0.3mm were excluded from these analyses. Cluster-State dFNC Indices: MDT = Mean Dwell Time. FDT = Fractional Dwell Time. NT = Number of Transitions. All analyses were two-tailed.

**Table F.1**

*Robust Linear Regression (MM): Moderation Analysis of the Role of Emotion Regulation Difficulties in the Association between Fractional Dwell Time in State 1 and Borderline Symptom Severity After Controlling for Age, Medication and Framewise Displacement*

**Model Summary – BSL-23 Total Score**

| **Model** | ***R*** | ***R²*** | **Adjusted *R²*** | **RMSE** | ***R²* Change** |
| --- | --- | --- | --- | --- | --- |
| M₀ | 0.330 | 0.110 | 0.001 | 19.744 | - |
| M₁ | 0.500 | 0.250 | 0.137 | 18.356 | 0.139 |

# Hierarchical Moderation Analysis -M0

| **Characteristic** | ***b*** | **95% CI (lower, upper)** | ***p*** |
| --- | --- | --- | --- |
| (Intercept) | 34.317 | 9.798, 58.835 | 0.008* |
| DERS | 0.363 | -0.057, 0.784 | 0.097 |
| FDT State 1 | 4.779 | -16.094, 25.652 | 0.655 |
| Age | 0.403 | -0.689, 1.496 | 0.473 |
| FD | -1.517 | -70.659, 67.625 | 0.965 |
| Medication | 0.422 | -5.548, 6.393 | 0.890 |

# Hierarchical Moderation Analysis-M1

| **Characteristic** | ***b*** | **95% CI (lower, upper)** | ***p*** |
| --- | --- | --- | --- |
| (Intercept) | 30.827 | 5.291, 56.363 | 0.022* |
| DERS | 0.382 | 0.058, 0.702 | 0.025* |
| FDT State 1 | 18.438 | -1.426, 38.302 | 0.076 |
| Age | 0.425 | -0.772, 1.622 | 0.490 |
| FD | 3.023 | -63.538, 69.585 | 0.929 |
| Medication | 0.075 | -2.413, 2.565 | 0.952 |
| DERS * FDT State 2 | -1.650 | -2.635, -0.665 | 0.002* |

*Note.* Robust linear regression models were estimated using MM-estimation with Tukey’s bisquare influence function. Reported p values and 95% confidence intervals are based on Wald tests using robust standard errors. FDT State 1 and DERS represent mean centered values. * indicates p < 0.05. M₀ = mean centered FDT State 1, mean centered DERS, M₁ = mean centered FDT State 1, mean centered DERS, mean centered FDT State 2 : mean centered DERS. DERS = Difficulty in Emotion Regulation Scale. FDT = Fractional Dwell Time. FD = Framewise Displacement. All models controlled for age, medication and framewise displacement.

**Table F.2**

*Robust Linear Regression (MM): Moderation Analysis of the Role of Childhood Trauma in the Association between Fractional Dwell Time in State 2 and Borderline Symptom Severity After Controlling for Age, Medication and Framewise Displacement*

| **Model** | ***R*** | ***R²*** | **Adjusted *R²*** | **RMSE** | ***R²* Change** |
| --- | --- | --- | --- | --- | --- |
| M₀ | 0.331 | 0.109 | 0.001 | 21.369 | - |
| M₁ | 0.517 | 0.267 | 0.157 | 19.700 | 0.158 |

# Model Summary – BSL-23

# Hierarchical Moderation Analysis -M0

| **Characteristic** | ***b*** | **CI (lower, upper)** | ***p*** |
| --- | --- | --- | --- |
| (Intercept) | 41.997 | 16.856, 67.138 | 0.002* |
| CTQ-SF | 0.257 | -0.199, 0.714 | 0.275 |
| FDT State 2 | -19.394 | -50.246, 11.456 | 0.224 |
| Age | 0.135 | -1.051, 1.323 | 0.823 |
| FD | 1.782 | -72.831, 76.395 | 0.962 |
| Medication | -0.051 | -4.701, 4.598 | 0.982 |

# Hierarchical Moderation Analysis-M1

| **Characteristic** | ***b*** | **95% CI (lower, upper)** | ***p*** |
| --- | --- | --- | --- |
| (Intercept) | 44.908 | 18.900, 70.917 | 0.001* |
| CTQ-SF | 0.041 | -0.398, 0.481 | 0.854 |
| FDT State 2 | -4.530 | -29.849, 20.787 | 0.727 |
| Age | 0.162 | -1.010, 1.335 | 0.787 |
| FD | -20.843 | -103.451, 61.763 | 0.623 |
| Medication | 2.222 | -2.282, 6.727 | 0.339 |
| CTQ-SF * FDT State 2 | -2.417 | -4.348, -0.486 | 0.018* |

*Note.* Robust linear regression models were estimated using MM-estimation with Tukey’s bisquare influence function. Reported p values and 95% confidence intervals are based on Wald tests using robust standard errors. FDT State 2 and CTQ-SF represent mean centered values. * indicates *p* < 0.05. M₀ = mean centered FDT State 2, mean centered CTQ-SF, M₁ = mean centered FDT State 2, mean centered CTQ-SF, mean centered FDT State 2 : mean centered CTQ-SF. CTQ-SF = Childhood Trauma Questionnaire – Short Form. FDT = Fractional Dwell Time. FD = Framewise Displacement. All models controlled for age, medication and framewise displacement.

**Table G.**

*Brief Descriptive Labels for Each Cluster-State*

| **State 1** | Weakly connected state with low integration |
| --- | --- |
| **State 2** | Moderately connected state with relatively stronger integration |
| **State 3** | Strongly connected state with high segregation |
